# 3-Phosphoinositide-dependent kinase 1 drives acquired resistance to osimertinib

**DOI:** 10.1038/s42003-023-04889-w

**Published:** 2023-05-11

**Authors:** Ismail M. Meraz, Mourad Majidi, Bingliang Fang, Feng Meng, Lihui Gao, RuPing Shao, Renduo Song, Feng Li, Yonathan Lissanu, Huiqin Chen, Min Jin Ha, Qi Wang, Jing Wang, Elizabeth Shpall, Sung Yun Jung, Franziska Haderk, Philippe Gui, Jonathan Wesley Riess, Victor Olivas, Trever G. Bivona, Jack A. Roth

**Affiliations:** 1 Department of Thoracic and Cardiovascular Surgery, The University of Texas MD Anderson Cancer Center, Houston, TX USA; 2 Department of Biostatistics, The University of Texas MD Anderson Cancer Center, Houston, TX USA; 3 Department of Biostatistics, Graduate School of Public Health, Yonsei University, Seoul, Korea; 4 Department of Bioinformatics and Computational Biology, The University of Texas MD Anderson Cancer Center, Houston, TX USA; 5 Department of Stem Cell Transplantation, The University of Texas MD Anderson Cancer Center, Houston, TX USA; 6 Department of Biochemistry, Baylor College of Medicine, Houston, TX USA; 7 Department of Medicine, University of California, San Francisco, San Francisco, CA USA; 8 Helen Diller Family Comprehensive Cancer Center, University of California, San Francisco, San Francisco, CA USA; 9 Department of Cellular and Molecular Pharmacology, University of California, San Francisco, San Francisco, CA USA; 10 University of California Davis Comprehensive Cancer Center, Sacramento, CA USA

**Keywords:** Non-small-cell lung cancer, Cancer therapeutic resistance

## Abstract

Osimertinib sensitive and resistant NSCLC NCI-H1975 clones are used to model osimertinib acquired resistance in humanized and non-humanized mice and delineate potential resistance mechanisms. No new EGFR mutations or loss of the EGFR T790M mutation are found in resistant clones. Resistant tumors grown under continuous osimertinib pressure both in humanized and non-humanized mice show aggressive tumor regrowth which is significantly less sensitive to osimertinib as compared with parental tumors. 3-phosphoinositide-dependent kinase 1 (PDK1) is identified as a potential driver of osimertinib acquired resistance, and its selective inhibition by BX795 and CRISPR gene knock out, sensitizes resistant clones. In-vivo inhibition of PDK1 enhances the osimertinib sensitivity against osimertinib resistant xenograft and a patient derived xenograft (PDX) tumors. PDK1 knock-out dysregulates PI3K/Akt/mTOR signaling, promotes cell cycle arrest at the G1 phase. Yes-associated protein (YAP) and active-YAP are upregulated in resistant tumors, and PDK1 knock-out inhibits nuclear translocation of YAP. Higher expression of PDK1 and an association between PDK1 and YAP are found in patients with progressive disease following osimertinib treatment. PDK1 is a central upstream regulator of two critical drug resistance pathways: PI3K/AKT/mTOR and YAP.

## Abstract (web summary)

Acquired resistance to osimertinib is driven by PDK1 in preclinical models of non-small cell lung cancer and reveals PDK1 as a potential target for restoring osimertinib sensitivity.

## Introduction

Tyrosine kinase inhibitors (TKIs) targeting the epidermal growth factor receptor (EGFR) have become the standard of care for NSCLC patients with EGFR driver mutations^1,2^. Osimertinib (AZD9291) is the first FDA-approved third-generation EGFR TKI, which irreversibly binds to EGFR proteins with T790M drug resistance mutations^3–6^. Not all patients respond initially, and responses, when they occur, are variable, typically incomplete, and temporary due to acquired drug resistance^7–15^. This obstacle to long-term patient survival highlights the need to identify acquired resistance mechanisms. Acquired drug resistance is a complex problem as multiple downstream effector proteins in bypass pathways can drive tumor regrowth, progression, and ultimately drug resistance^16–19^.

The PI3K/AKT/mTOR has been implicated in NSCLC acquired resistance, but the role of AKT-independent signaling downstream of PI3K is not well-characterized^20,21^. One protein that transduces PI3K signaling, is the serine/threonine kinase 3-phosphoinositide-dependent protein kinase 1 (PDK1 also known as PDPK1). PDK1 is a pleotropic regulator of 60 serine/threonine kinase proteins classified into 14 families of the AGC kinase superfamily^22^. It has multifunctional oncogenic activity, concurrently activating pro-survival protein kinases^23,24^, and suppressing apoptosis in lung cancer^25^. PDK1 is also implicated in tumors driven by KRAS genetic alterations, and regulates immune cell development, including T and B cells, and their functions in the tumor microenvironment (TME)^26–29^. NSCLC sera compared to healthy samples were reported to have significantly higher levels of PDK1 mRNA expression^30^. Recently, PDK1 started to gain a wide interest as a drug target, which has so far led to the filing of more than 50 patents^24^.

In this study we provide evidence that (a) PDK1 is a potential driver of osimertinib acquired resistance, (b) PDK1 genetic and pharmacological targeting restores osimertinib response in resistant clones and their derived human xenografts and PDXs, (c) PDK1 knock-out dysregulates PI3K/AKT/mTOR signaling and YAP activation, and (d) patient biopsies from EGFR mutant lung adenocarcinoma tumors with the highest PDK1 & YAP expression are found in a subset of patients with progressive disease following TKI treatment, suggesting they could be responsive to PDK1 inhibitors.

## Results

### Development of osimertinib resistance in NCI-H1975-OsiR cells

**Fig. 1:**
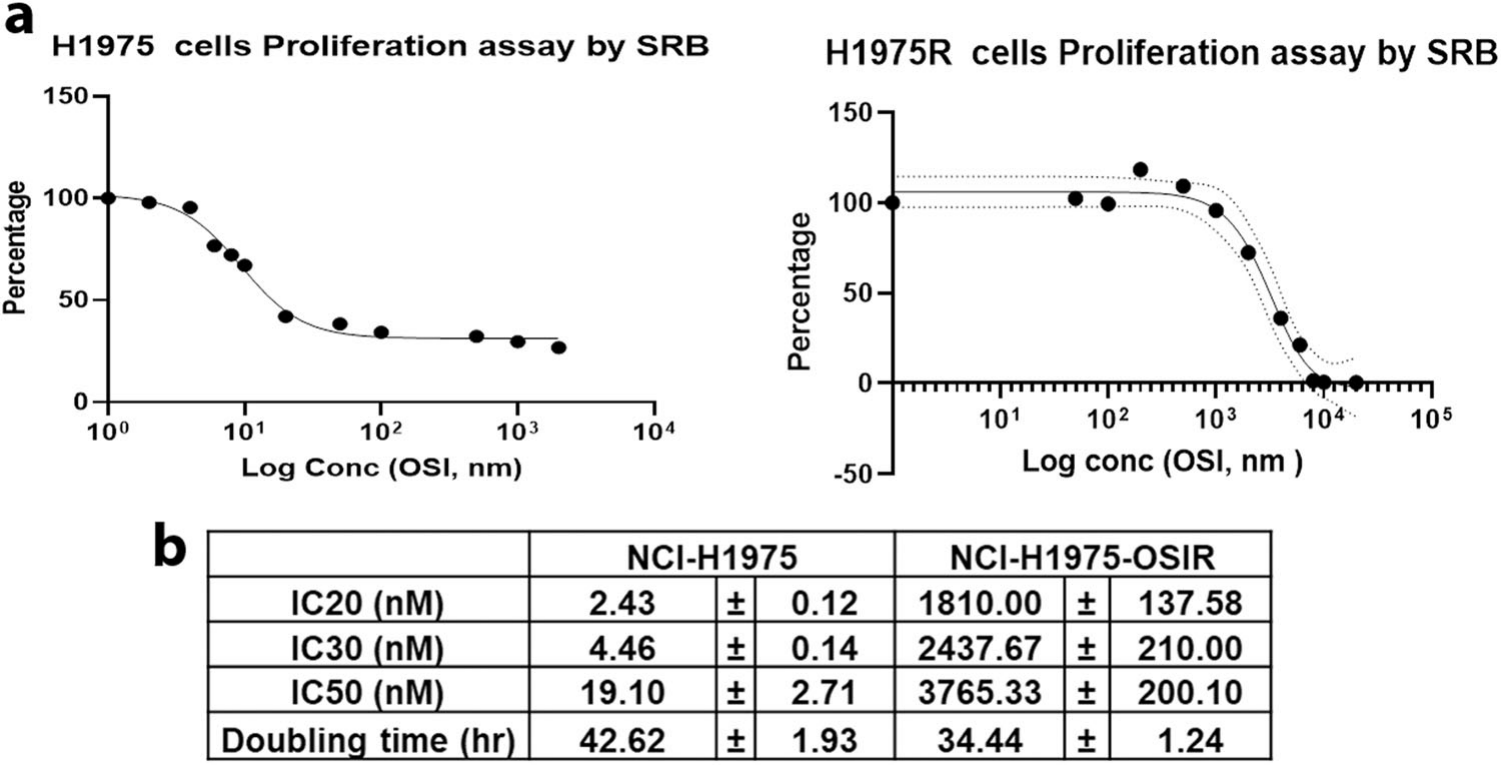
Effect of osimertinib on survival of NCI-H1975 and NCI-H1975-OsiR cells. **a** SRB assay was performed using two sets of osimertinib concentrations for sensitive and resistant cells to determine the IC_20_, IC_30_ and IC_50_. **b** Table shows the IC_20_, IC_30_, and IC_50_ values of H1975 and H1975-OsiR cells and the cell doubling time. Data shown represent the mean ± SE of three independent experiments.

The human NSCLC NCI-H1975 cell line harbors two EGFR point mutations, T790M and L858R, in exons 20 and 21, respectively and is highly sensitive to osimertinib^31^. In order to study acquired resistance, osimertinib-resistant cell line, NCI-H1975-OsiR, was developed by continuous exposure of osimertinib through dose escalation (0.5–2.5 µM) to the NCI-H1975 cells until the emergence of the osimertinib-resistant clone, NCI-H1975/OSIR. The resistant cells have less longitudinal cell morphology and a faster doubling time than the parental-sensitive clone (34 h vs. 42 h; Fig. 1b). An osimertinib sensitivity assay using Sulforhodamine B (SRB) to measure cell proliferation showed that NCI-H1975-OsiR cells were approximately 200-fold more resistant to osimertinib than their NCI-H1975 counterparts, as shown by IC 20, 30, and 50 values (Fig. 1). The drug resistance index in NCI-H1975-OsiR cells was 197.14, which was much higher than 10 and indicates the cell line is osimertinib resistant^32,33^.

### No new EGFR mutations or loss of the EGFR L858R and T790M mutations found in NCI-H1975-OsiR

Whole-exome sequence (WES) analysis was performed to identify any acquired or lost EGFR mutations during osimertinib treatment in the entire protein-coding regions of the genome of the NCI-H1975-OsiR clone. The results showed there were no new EGFR mutations or loss of the EGFR L858R and T790M mutations. However, there were 37 new exonic mutations, including 0 indels, 27 nonsynonymous single nucleotide variants (SNV), 4 stopgain, and 6 synonymous mutations (Suppl Fig. 1A). EGFR and pEGFR (Tyr1173) expression were also checked in this EGFR mutant osimertinib-sensitive and resistant isogeneic cell lines by western blot. Total EGFR expression was increased with osimertinib treatment in NCI-H1975-parental cells and the level of EGFR in NCI-H1975-OsiR is comparable with the level in osimertinib treated NCI-H1975 cells (Suppl Fig. 1B). Osimertinib treatment was able to completely shut down the phosphorylation of EGFR in sensitive cells, but very low level or no phospho-EGFR (Tyr1173) expression was found in osimertinib maintained NCI-H1975-OsiR cells (Suppl Fig. 1B). The list of these new mutations and their projected pathways are shown in supplemental Fig. 1a, c.

### Development of osimertinib resistance in NCI-H1975-OsiR xenografts in NSG mice and alteration of protein expression in resistant tumors

Osimertinib-resistant NCI-H1975-OsiR xenograft tumors were developed in NSG mice under continuous osimertinib pressure according to the treatment strategy shown in Fig. 3a. In contrast to the in vitro cell line growth (Fig. 1b), NCI-H1975-OsiR tumors grew much slower than NCI-H1975-parental tumors (Fig. 3b). But similar to resistant cells in vitro, NCI-H1975-OsiR xenograft tumors were significantly less sensitive to osimertinib (Fig. 3c–e). A dose-dependent osimertinib response was found in NCI-H1975-parental tumors (Fig. 3c, e), which was completely lost in NCI-H1975-OsiR tumors (Fig. 3d, e). Releasing the osimertinib pressure during NCI-H1975-OsiR tumor growth increased the osimertinib sensitivity. Residual tumors from osimertinib-sensitive and resistant xenografts were harvested and protein expression analysis was performed by Reverse-Phase Protein Array (RPPA) analysis. The results showed that expression of a set of immune-related proteins was altered in resistant tumors compared with sensitive tumors. STING, Macrophage Inhibitory Factor (MIF), CD20, B7-H3 (co-stimulatory molecule for T cells), HMHA1 (Minor histocompatibility complex) were significantly downregulated and HLA-DQA1 was upregulated in osimertinib-resistant tumors compared to sensitive tumors (Suppl Fig. 2A). Expression of another set of immune-related proteins significantly changed after prolonged osimertinib treatment in NCI-H1975 tumors (Suppl Fig. 2B). Immune activation related proteins including HMHA1, B7-H3, Granzyme-B, MIF, CD20, PD-1, B7-H4 were significantly downregulated, while HLA-DQA1 and PD-L1 were upregulated, whereas a set of metabolic proteins including Glutaminase, Pdcd4, Glutamate, PDHA1, GCLC, FGF-basic were significantly upregulated after continuous treatment of osimertinib (Suppl Fig. 2B). These alterations confirm acquisition of an immunosuppressive phenotype, which is consistent with clinical observations of EGFR tumors with acquired drug resistance thus confirming the clinical relevance of this model^34^.

### Modeling osimertinib acquired resistance in the humanized mouse model

**Fig. 2:**
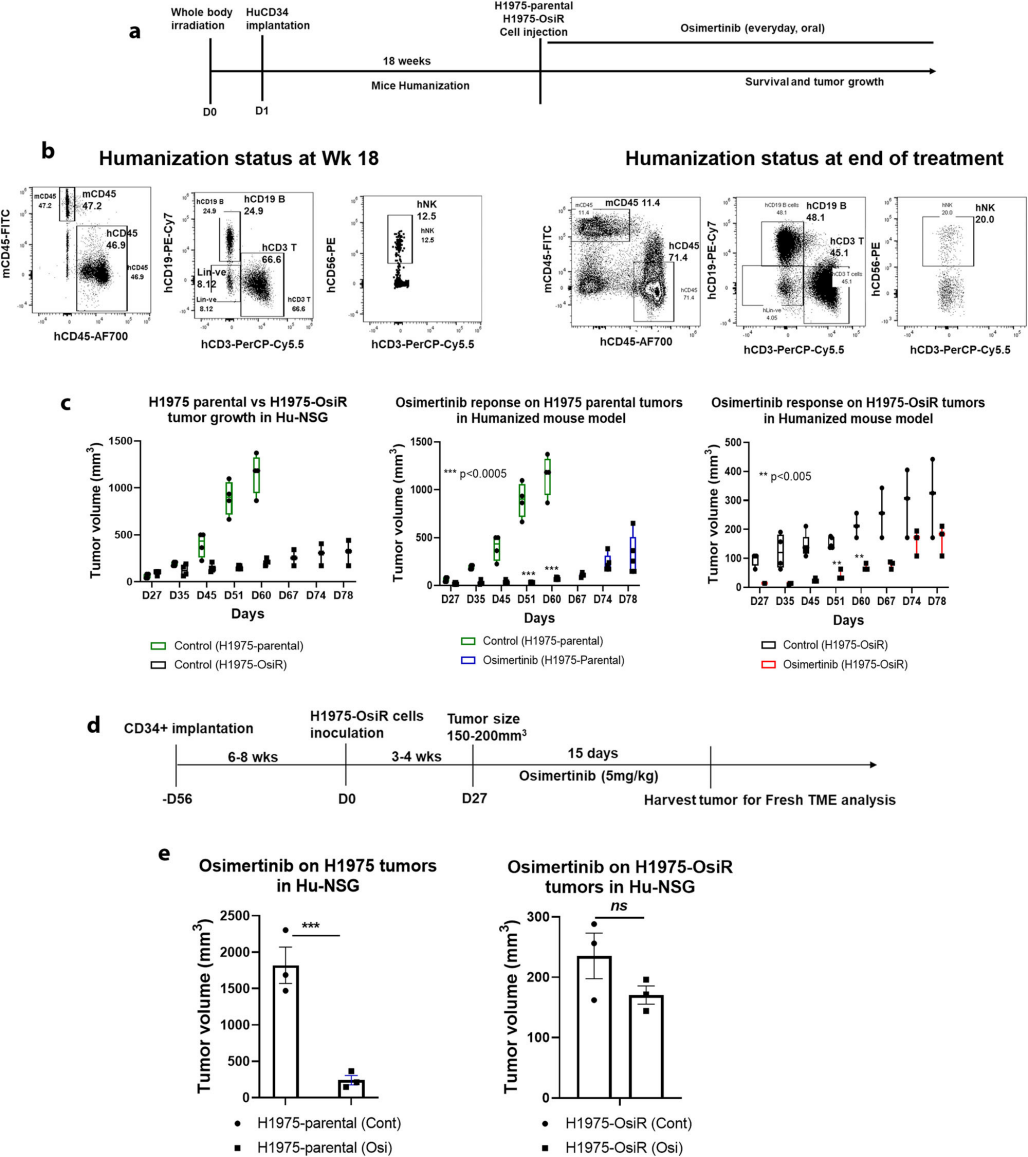
Effect of osimertinib on humanized H1975 and H1975-OsiR xenografts. Humanized mice were generated by human CD34 stem cells implantation. After mice become humanized with over 25% human CD45 cells, the NCI-H1975 and NCI-H1975-OsiR cells were injected subcutaneously. **a** Experimental strategy for mouse humanization, tumor cell inoculation, and osimertinib prolonged treatment, **b** Levels of human immune cell repopulation in humanized mice at different time points. **c** Tumor growth comparison between NCI-H1975-parental vs. NCI-H1975-OsiR and the effect of osimertinib on their growth. **d** Experimental strategy for mouse humanization, tumor cell inoculation, and osimertinib short term treatment. **e** Antitumor effect of osimertinib on tumor growth. In each experiment, *N* ≥ 5 humanized mice/group were used. The humanized mice experiments were repeated *N* = 3 times.

**Fig. 3:**
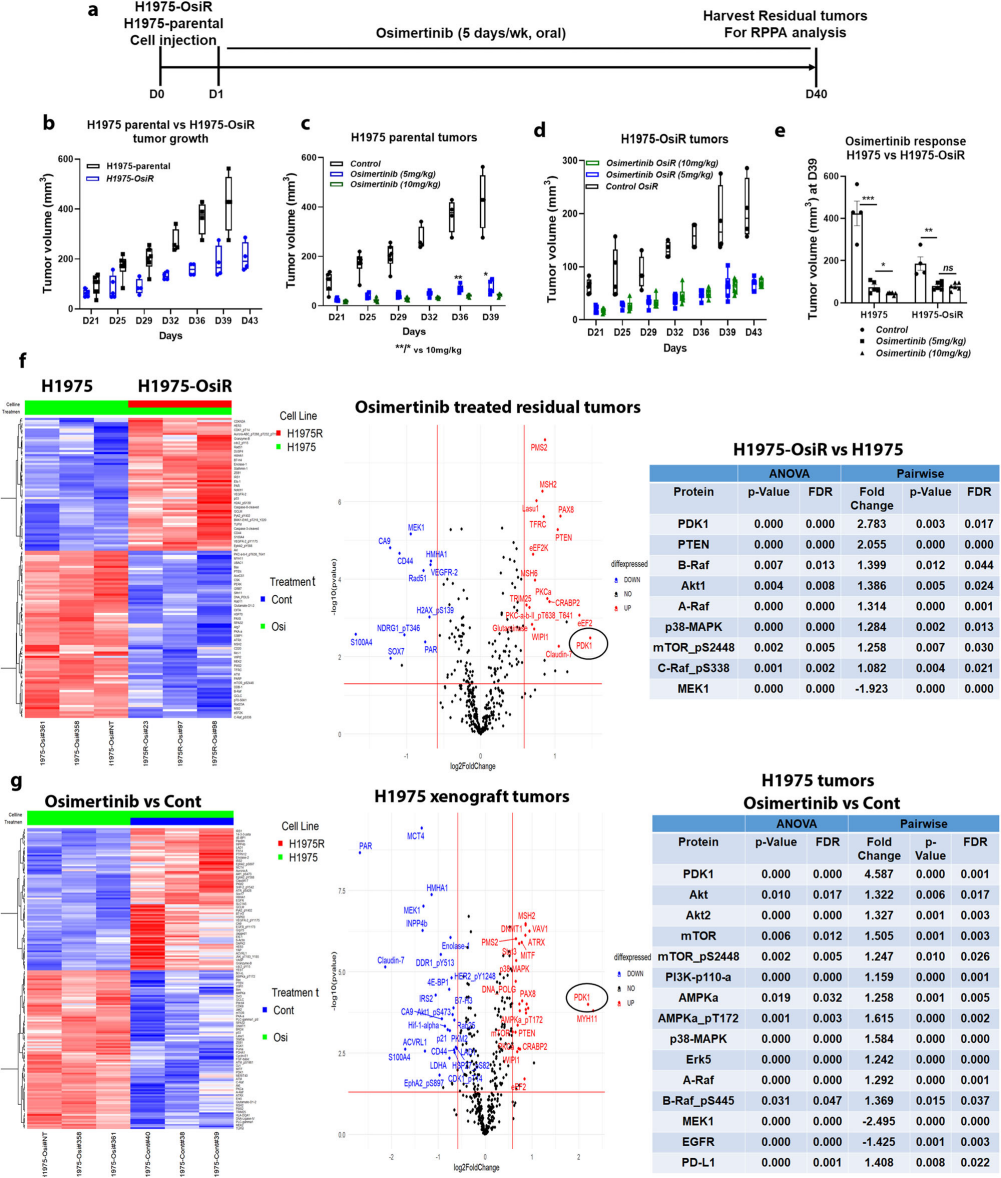
RPPA Gene expression profile analysis in osimertinib treated residual xenograft tumors. NCI-H1975 and NCI-H1975-OsiR xenograft tumors were developed in NSG mice. Osimertinib-resistant tumors were developed under continuous osimertinib pressure by treating the mice with osimertinib following tumor cell implantation. At the end of the treatment, the residual tumors were harvested and snap-frozen for RPPA analysis. **a** Treatment strategy shows the timeline for osiemretinib treatment and tumor harvest for RPPA, **b** Growth curve comparison between NCI-H1975 and NCI-H1975-OsiR tumors. **c** Effect of osimertinib on NCI- H1975 parental tumors (*N* = 6mice/group) at two different doses; 5 mg/kg and 10 mg/kg, **d** Effect of osimertinib on NCI-H1975-OsiR tumors (*N* = 6mice/group) at two different doses; 5 mg/kg and 10 mg/kg, **e** Osimertinib response at D39, **f** Pairwise comparison between NCI-H1975 and NCI-H1975-OsiR residual tumors after osimertinib treatment, left shows the heatmap, middle shows the volcano plot and right has the list of upregulated proteins in NCI-H1975-OsiR as compared with NCI-H1975 xenografts. **g** Heatmap (left), volcano plot (middle) show the differences in protein expression in NCI-H1975 residual tumors after prolonged osimertinib treatment vs. control tumors. The list of major upregulated proteins are shown on the right. The criteria of protein selection for significantly up- down-regulation were: 1. Significant in overall *F*-test (FDR-adjusted *P*-value < 0.05); 2. Significant in pairwise comparison. (FDR-adjusted *P*-value < 0.05); 3. The fold-change of >1.5 or < −1.5 indicates whether a gene is upregulated or downregulated.

We investigated whether this model of acquired drug resistance maintained the resistant phenotype in the presence of human tumor microenvironment. The major limitation of current experimental rodent models is that many functional aspects of human innate and adaptive immunity cannot be recapitulated with non-humanized mouse models. Our improved humanized mouse model is better suited to model osimertinib acquired resistance and provides insight into the complex interaction of osimertinib with variable contextures of the tumor microenvironment (TME)^35^. NCI-H1975 and NCI-H1975-OsiR xenografts were implanted with fresh CD34^+^-derived humanized mice developed from different donors with partial HLA compatibility. The humanization protocol, the levels of human immune reconstitution in humanized mice, growth characterization of tumor xenografts and osimertinib treatment are illustrated in Fig. 2a–c. The level of human CD45^+^ and reconstituted T, B, and NK cells before the tumor implantation at wk 18 and after the experiment are shown in Fig. 2b, and are 2–3-fold higher than the general standard for mouse humanization, which is a minimum level of 25% of reconstituted human CD45 cells. Flow cytometry gating strategy was shown in Suppl Fig 11. Results from two independent experiments with long (78 days) (Fig. 2a, c) and short term (27 days) (Fig. 2d, e) osimertinib treatment (5 mg/kg), showed that growth of NCI-H1975 tumors were significantly inhibited in both experiments. In contrast, the inhibition of NCI-H1975-OsiR tumor growth with osimertinib treatment was statistically insignificant (Fig. 2c; right panel & Fig. 2e; right panel) showing initial growth stabilization followed, after a short time, by tumor regrowth. Because NCI-H1975-OsiR xenograft tumors were developed under constant osimertinib (5 mg/kg) pressure, twenty-four hours post implantation, their growth was slower than their untreated NCI-H1975 counterparts. Like humanized mice in Fig. 2c, the growth rate of osimertinib-resistant NCI-H1975-OsiR xenograft tumor was significantly slower than that of NCI-H1975 parental tumors (Fig. 3b).

### Upregulation of PDK1, 3-phosphoinositide-dependent kinase 1, in NCI-H1975-OsiR xenografts

**Fig. 4:**
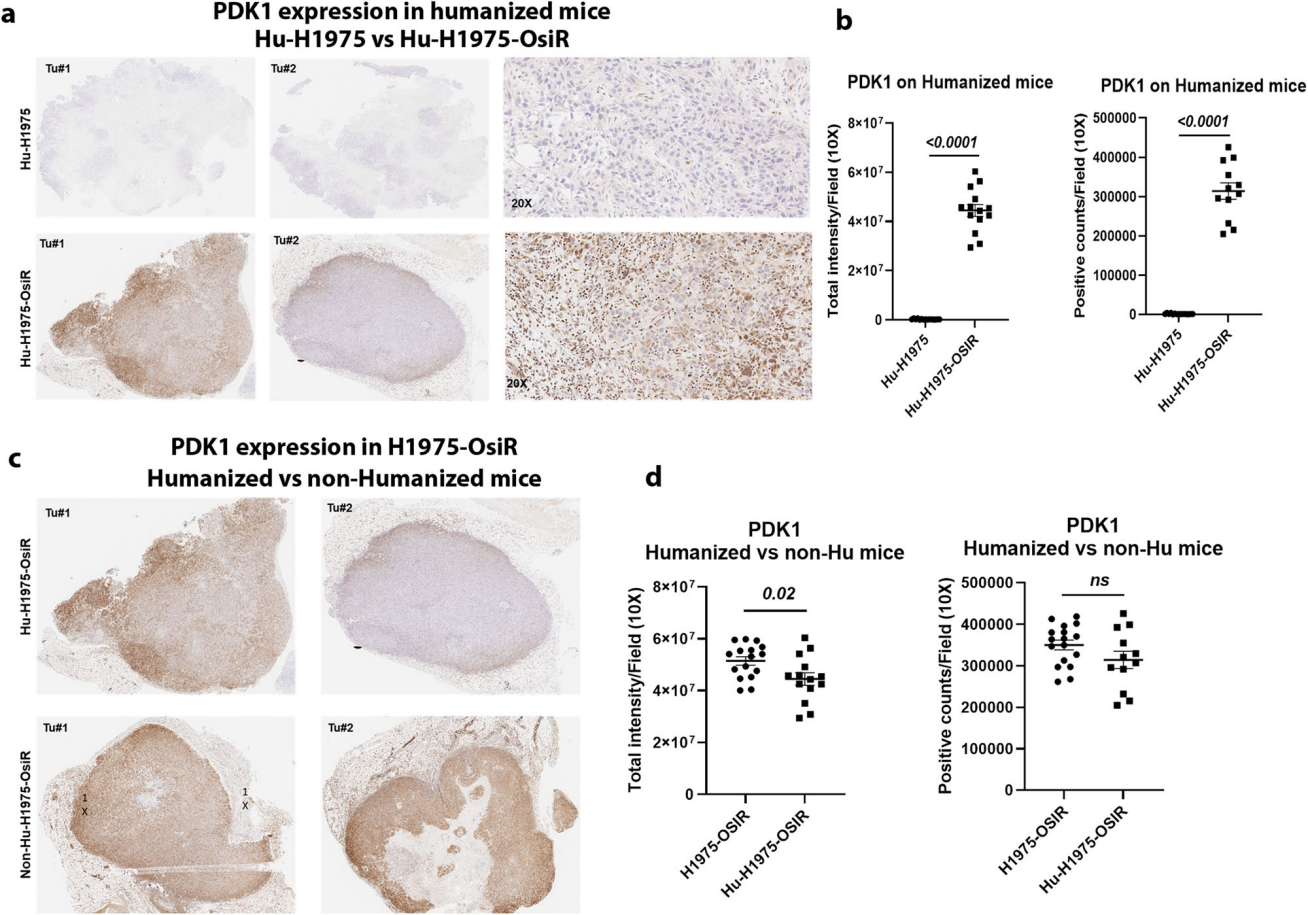
PDK1, 3-Phosphoinositide-dependent kinase 1, was upregulated in osimertinib-resistant xenograft tumors developed in humanized mice. NSG mice were humanized by human CD34 stem cells implantation, after humanization, NCI-H1975 and NCI-H1975-OsiR cells were injected subcutaneously. The resistant xenograft tumors were developed under osimertinib pressure throughout the experiment. At the end, tumors were harvested and IHC was performed for PDK1 expression. **a** the level of PDK1 expression between Hu-H1975 and Hu-H1975-OsiR tumors; **b** the PDK1 signal intensity and PDK1-positive cell counts were quantitated; **c** the level of PDK1 expression was compared in NCI-H1975-OsiR tumors developed in humanized and non-humanized mice; **d** the signals were quantitated, and statistics were performed.

To understand the underlying mechanism of osimertinib acquired resistance, NCI-H1975 and NCI-H1975-OsiR xenografts were grown in NSG mice untreated or treated with osimertinib. Tumors were grown under constant osimertinib pressure according to the treatment strategy shown in Fig. 3a. Untreated and osimertinib treated residual tumors were harvested for proteomic analysis using an antibody‐based functional proteomic analysis, RPPA, consisting of a 400 antibody panel, which includes serine/threonine and tyrosine kinases. Heat map analysis of pairwise comparison of protein expression profiles between NCI-H1975 and NCI-H1975-OsiR osimertinib treated residual tumors (Fig. 3f), and NCI-H1975-OsiR osimertinib treated and untreated groups (Suppl Fig. 3) showed upregulation of two distinct protein signatures. A common set of proteins including B-Raf, A-Raf, C-Raf, AKT, mTOR, p38-MAPK, and Erk5, which are associated with tumor growth and proliferation were upregulated in osimertinib treated NCI-H1975-OsiR tumors (Fig. 3f, right; Suppl Fig. 3c). EGFR_pY1173, MEK1 was significantly downregulated in osimertinib treated tumors (Suppl Fig. 3C; Fig. 3f, right). Interestingly in the signatures in both pairwise comparisons, PDK1 was a highly significant outlier and upregulation was the highest in the osimertinib-resistant tumors (Fig. 3f, middle; Suppl Fig. 3B). In the former pairwise comparison, the PDK1 expression level increased by 2.783-fold (Fig. 3f, middle), and in the latter by 2.4-fold (Suppl Fig. 3B). This suggests that PDK1 differential expression between NCI-H1975 and NCI-H1975-osiR might play a potential role in the latter’s acquisition of resistance to osimertinib. PDK1 regulates a number of serine/threonine protein kinases of the AGC kinase superfamily, activating multiple pro-survival and oncogenic pathways, and suppressing apoptosis in lung cancer^22–25^. NCI-H1975 parental tumors were treated with osimertinib for a prolonged period (Fig. 3a). The sensitive tumors showed significant sensitivity towards osimertinib as compared with no treatment (Fig. 3c). We analyzed the residual tumors using RPPA. The heatmap showed that a distinct set of proteins were significantly upregulated and another set downregulated (Fig. 3g). Similar to the osimertinib-resistant tumors, the volcano plot showed the PDK1 protein expression was increased significantly (more than 4.5-fold as compared with control tumors) in residual tumors (Fig. 3g, middle). EGFR was downregulated, but PI3K_p110-a, AKT, AKT2, mTOR, mTOR_pS2448 were significantly upregulated. AMPKa, AMPKa_pT172, and raf proteins were upregulated whereas MEK1 was downregulated in osimertinib treated residual tumors (Fig. 3g, right). This data suggested that PI3K/AKT/mTOR pathway is activated in osimertinb treated residual cells. We performed IHC to visualize the expression level of PDK1 in osimertinib-resistant tumors (NCI-H1975-OsiR vs. NCI-H1975) developed in humanized and non-humanized mice. PDK1 expression was significantly upregulated in Hu-H1975-OsiR tumors as compared with Hu-H1975 tumors (Fig. 4a, b), which was consistent with the RPPA analysis in non-humanized mice (Fig. 3). We also compared the level of PDK1 expression between humanized vs. non-humanized mice. A very high level of PDK1 expression was found in osimertinib-resistant tumors developed in both humanized and non-humanized mice (Fig. 4c, d).

### Mass spectrometry-based proteomic analysis confirms significant upregulation of PDK1 and dysregulation of downstream signaling in osimertinib-resistant NCI-H1975-OsiR clones

To further investigate the role of PDK1 in mediating osimertinib acquired resistance, as suggested by RPPA analysis and IHC in humanized and non-humanized mice, we profiled proteins in the global and phospho-proteome of NCI-H1975 and NCI-H1975-OsiR isogenic clones along with PDK1 knockout (NCI-H1975-OsiR-PDK1^-/-^) and PDK1 restored (NCI-H1975-OsiR-PDK1^++/++^) clones using an unbiased robust mass spectrometry (MS)-based proteomics workflow^26,27^. Global and phospho-proteomic analyses covered over 8000 gene protein products (GPS), and over 4000 GPS, respectively. After label-free nanoscale liquid chromatography coupled to tandem mass spectrometry (nanoLC-MS/MS) analysis using a Thermo Fusion Mass spectrometer, the data was processed and quantified against NCBI RefSeq protein databases in a Proteome Discover 2.5 interface with Mascot search engine (Saltzman, Ruprecht). The dendrogram clustering on four isogenic cell lines (NCI-H1975, NCI-H1975-OsiR, NCI-H1975-OsiR-PDK1^-/-^ and NCI-H1975-OsiR-PDK1^++/++^) with three biological replicates showed that the parental cells and PDK1 KO cells belonged into one cluster whereas osimertinib-resistant cells and NCI-H1975-OsiR-PDK1^++/++^ cells were together into another cluster (Suppl Fig. 4A). This indicated that PDK1 KO transformed the resistant cells into sensitive phenotypes. Similarly, two components curve of phospho-proteomic data of four isogeneic cell lines showed that all three replicates on each cell line were clustered together. Two component curves also separated all samples into two major groups where the sensitive NCI-H1975 cells and PDK1 KO cells remained on one side of the component curve and the NCI-H1975-OsiR and PDK1 rescued cells were on other side of the curve indicating the homology of PDK KO cells to osimertinib-sensitive cells (Suppl Fig. 4B). The heat map showed that a distinct set of phospho-proteins were upregulated, and another set of phospho-proteins were downregulated in NCI-H1975-OsiR cells compared to parental NCI-H1975 (Suppl Fig. 4C). The level of pPDK1 in NCI-H1975-OsiR, was 14-fold higher than in the NCI-H1975 osimertinib-sensitive cells (Suppl Fig. 4D). These results are compatible with those of the RPPA, validating PDK1 differential expression and activity between NCI-H1975 and NCI-H1975-OsiR cells and identifying PDK1 as a potential driver of osimertinib acquired resistance. Consistent with the RPPA data in Fig. 3, enrichment analysis of MS-phospho-proteomics found that upregulation of PDK1 in osimertinib-resistant cells, which is the upstream kinase of growth promoting pathways, was also associated with the enrichment of PI3K/AKT/mTOR and mTORC1 signaling (Suppl Fig. 4E, F). The findings of activation of the AKT/mTOR pathway was consistent with a recent Mass Spec analysis of osimertinib-resistant cell lines^36^. Moreover, the enrichment of these gene sets linked with these two signaling pathways were downregulated when PDK1 was knocked out in NCI-H1975-OsiR cells (Suppl Fig. 4E, F).

### Pharmacological inhibition and genetic knock-out of PDK1 sensitizes NCI-H1975-OsiR clones to osimertinib

**Fig. 5:**
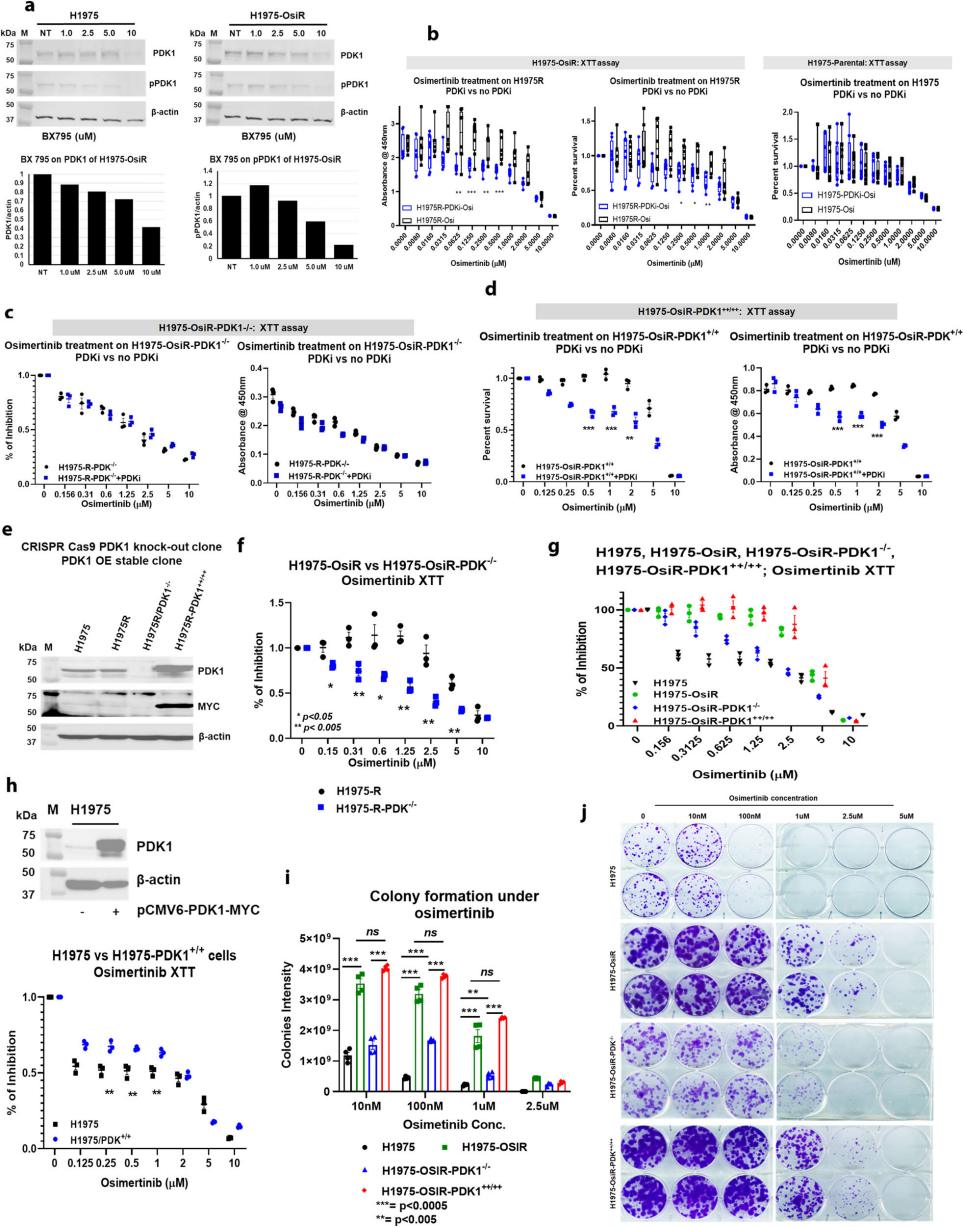
In vitro inhibition of PDK1 or PDK1 knockout increased osimertinib sensitivity and inhibited colony formation. **a** NCI-H1975 and NCI-H1975-OsiR cells were treated with PDKi, BX 795, and dose-dependent inhibition of PDK1 and pPDK1 are shown; **b** osimertinib XTT was performed on NCI-H1975 and NCI-H1975-OsiR cells in the presence or absence of BX 795 and shows that BX795 renders NCI-H1975-OsiR sensitive to osimertinib; **c** osimertinib XTT with or without BX 795 on PDK1 knockout clone, NCI-H1975-OsiR-PDK1^-/-^; **d** osimertinib XTT on re-expressing PDK1 into PDK1 KO clone, NCI-H1975-OsiR-PDK1^++/++^ in presence or absence of BX 795. **e** Generation of CRISPR-cas9 mediated PDK1 knockout clone, NCI-H1975-OsiR-PDK1^-/-^ and rescue of PDK1 in the KO clone by stably expressing the PDK1 plasmid to make NCI-H1975-OsiR-PDK1^++/++^ clones; **f** XTT assay shows the effect of PDK1 KO on osimertinib sensitivity; **g** XTT shows rescue of PDK1 expression in the PDK1 KO clone reverted the resistance to osimertinib. **h** Transient expression of PDK1 into NCI-H1975-parental cells and osimertinib XTT on transiently expressing PDK1 cells; **i**, **j** Colony formation assay shows osimertinib differential sensitivity among all clones. Data shown represent the mean ± SE of three independent experiments.

We validated the functional role of PDK1 in osimertinib acquired resistance in vitro and in vivo. NCI-H1975-OsiR clones were left untreated, treated with osimertinib, the PDK1-selective inhibitor BX-795, or the combination of both, and assayed for survival by XTT assay. NCI-H1975 cells were used as controls. Figure. 5a showed dose-dependent inhibition of PDK1 and pPDK1 expression by BX795 in both isogenic clones by western blot analysis. XTT assay showed that the sensitivity of osimertinib towards NCI-H1975 parental cells did not change in the presence of BX 795, whereas it rendered NCI-H1975-OsiR sensitive to osimertinib, as shown by a significant increase in cell death (Fig. 5b). To functionally implicate PDK1 as a mediator of osimertinib acquired resistance and eliminate drug off-target effects, CRISPR-cas9 mediated PDK1 knockout (NCI-H1975-OsiR-PDK1^-/-^) clones and PDK1 rescue clones where PDK1 knockout cells stably re-expressed PDK1 (NCI-H1975-OsiR-PDK1^++/++^) were generated (Fig. 5e) and assayed for survival and colony formation following osimertinib treatment. PDK1 knockout cells were significantly sensitive to osimertinib as compared with NCI-H1975-OsiR (Fig. 5f), which is in consistent with the findings on PDK1 inhibition by BX 795 (Fig. 5b). Rescuing PDK1 in knockout cells reversed osimertinib resistance comparable to NCI-H1975-OsiR cells (Fig. 5g). We also tested the effect of BX 795 on NCI-H1975-OsiR-PDK1^-/-^ and NCI-H1975-OsiR-PDK1^++/++^ cells. Compared to the parental cells NCI-H1975, PDK1 knockout cells did not differ in osimertinib sensitivity (Fig. 5c), whereas NCI-H1975-OsiR-PDK1^++/++^ cells showed comparable sensitivity to osimertinib in the presence of BX 795 (Fig. 5d). We then transfected PDK1 into sensitive cells, which showed significantly increased resistance to osimertinib as compared with non-PDK1 transfected NCI-H1975 cells (Fig. 5h). In dose titration experiments, at nanomolar and micromolar osimertinib concentrations, PDK1 knockout cells (NCI-H1975-OsiR-PDK1^-/-^) had significantly reduced colony formation in the presence of osimertinib compared with NCI-H1975-OsiR cells (Fig. 5i, j). Rescuing PDK1 in knockout cells (NCI-H1975-OsiR-PDK1^++/++^) significantly increased colony formation similar to NCI-H1975-OsiR cells (Fig. 5i, j).

### In vivo inhibition of PDK1 enhances osimertinib response in resistant PDXs

**Fig. 6:**
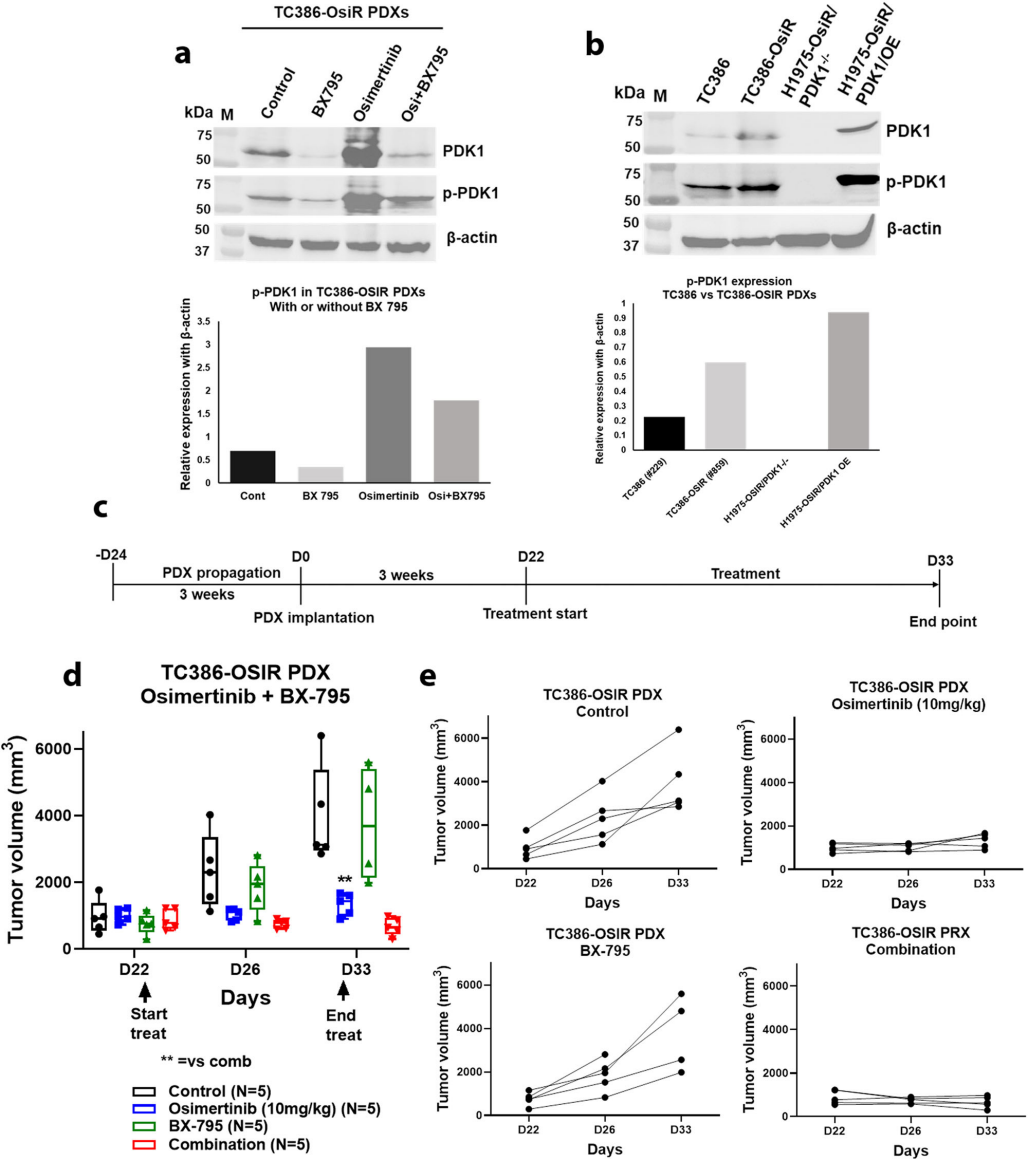
In vivo inhibition of PDK1 by PDK1 inhibitor, BX795, enhanced osimertinib response in resistant PDXs. Fresh osimertinib-resistant TC386-OsiR PDXs were implanted into NSG mice. When PDX sizes reached around 200mm^3^, PDXs bearing mice were randomized into different treatment arms for the treatment of osimertinib and BX 795. **a** End of the treatment harvested PDXs were checked for PDK1 and pPDK1 (S241) expression in TC386-OsiR PDXs treated with BX 795, osimertinib and osimertinib + BX 795. **b** Level of PDK1 and pPDK1 (S241) expression in osimertinib-sensitive TC386 PDX, and ositmertinib-resistant TC386-OsiR PDX tissues comparing with NCI-H1975-OsiR/PDK1^-/-^ and NCI-H1975-OsiR-PDK1^++/++^ cells, **c** Osimertinib + BX795 treatment strategies in osimertinib-resistant PDXs, **d** Antitumor activity of Osimertinib + BX795 combination on TC386-OsiR PDXs, **e** Growth curves of TC386-OsiR PDXs bearing individual mice in different treatment groups. Each treatment group was *N* = 5 PDX bearing mice.

We developed EGFR mutant osimertinib-sensitive and resistant TC386 isogenic PDXs. The parental TC386 PDX was highly sensitive to osimertinib (Suppl Fig. 5). The resistant TC386R PDX was generated through continuous treatment with osimertinib over a prolonged period of time and subsequent passages to four generations (Suppl Fig. 6A). A later generation (RG4) showed significantly more resistance than an earlier generation (RG1) without the acquisition or loss of EGFR mutations present in the parental PDX (Suppl Fig. 6B). Whole-exome sequencing of TC386-OsiR PDX showed that the EGFR (Del745-750) mutation remained same as the TC386 parental PDX with acquisition of additional mutations including SETD1B, MUC2, FAT3, EIF3M, HRCT1, RB1CC1 (Suppl Fig. 7A, B). When the PDK1 and pPDK1 level were compared between parental and resistant PDXs, higher levels of both PDK1 and pPDK1 were found in the TC386-OsiR PDX as compared with the parental TC386 PDX (Fig. 6b). To evaluate the antitumor effect of the PDK1 inhibitor (PDKi), BX 795 on resistant PDXs, we treated TC386-OsiR PDXs according to the treatment strategy shown in Fig. 6c treating with BX 795, osimertinib and the combination. The combination treatment inhibited the tumor growth drastically, which was statistically significant as compared to the single agents (Fig. 6d). Individual mouse PDX growth curves showed that tumors all regressed in the combination group compared to other groups (Fig. 6e). BX 795 greatly reduced PDK1 and pPDK1 expression in the BX 795 treated tumors as compared to untreated or osimertinib alone treated tumors (Fig. 6a). We assessed the effect of BX 795 (PDKi) in combination with osimertinib in the NCI-H1975-OsiR xenograft model, and an enhanced antitumor effect was found when BX 795 was combined with osimertinib (suppl Fig. 7C, D). IHC of PDK1 on the residual tumor tissues showed that the level of PDK1 expression was significantly lower in BX 795 treated tumors as compared with non-BX795 treated samples (Supplement Fig. 7E, F). Taken together, the in vitro and in vivo evidence support PDK1 as a driver of osimertinib acquired resistance in two independent models.

### PDK1 knock-out alters activation of the AKT/mTOR pathway

**Fig. 7:**
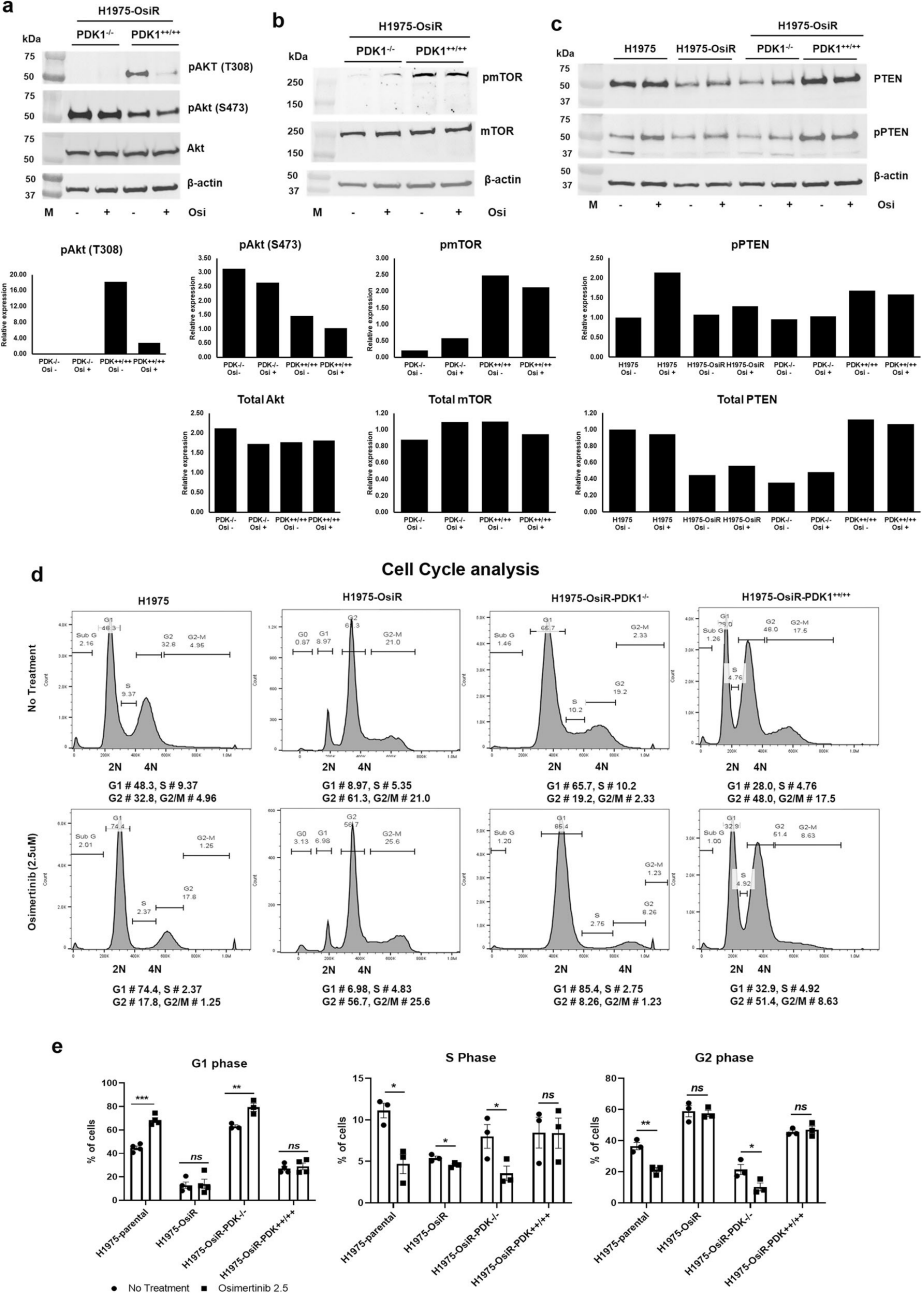
PDK1 knock-out dysregulates AKT/mTOR signaling and promotes cell cycle arrest. PDK1 KO cells, NCI-H1975-OsiR-PDK1^-/-^ and PDK1 re-expressing cells, NCI-H1975-OsiR-PDK1^++/++^ were treated with osimertinib and upstream and downstream signaling molecules were investigated by the western blot. **a** AKT, **b** mTOR, and **c** PTEN expression and its quantitation in NCI-H1975-OsiR-PDK1^-/-^ and NCI-H1975-OsiR-PDK1^++/++^ cells and alteration by osimertinib treatment. **c** PTEN expression in NCI-H1975-parental, NCI-H1975-OsiR, NCI-H1975-OsiR-PDK1^-/-^ and NCI-H1975-OsiR-PDK1^++/++^ cells. **d** Cell cycle analysis of NCI-H1975-parental, NCI-H1975-OsiR, NCI-H1975-OsiR-PDK1^-/-^ and NCI-H1975-OsiR-PDK1^++/++^ cells after osimertinib treatment. **e** Quantitation of cells in difference phases and its alteration by osimertinib treatment.

Activation of the oncogenic PI3K/AKT/mTOR pathway mediates tumorigenesis and resistance to EGFR TKIs in NSCLC^16–19^. Since PDK1 represents a pivotal node in this important signaling axis, we analyzed the phosphorylation status of its major signaling effectors in NCI-H1975, NCI-H1975-OsiR, NCI-H1975-OsiR-PDK1^-/-^ and NCI-H1975-OsiR-PDK1^++/++^ clones. Figure 7a shows that total AKT expression was similar in both NCI-H1975-OsiR-PDK1^-/-^ and NCI-H1975-OsiR-PDK1^++/++^ clones and osimertinib treatment had no effect on its level. Whereas, osimertinib significantly reduced AKT phosphorylation at the threonine 308 (T308) residue in the NCI-H1975-OsiR-PDK1^++/++^ clone (Fig. 7a), which is known to be the site activated by PDK1^23^. The level of pAKT (T308) was significantly reduced in NCI-H1975-OsiR-PDK1^-/-^ cells (Fig. 7a). The level of phosphorylation of AKT (S473) in NCI-H1975-OsiR-PDK1^-/-^ was higher than that of NCI-H1975-OsiR-PDK1^++/++^ cells, which can be catalyzed by multiple proteins but not PDK1 (Fig. 7a). Osimertinib had no effect on this activity. The level of total mTOR expression was similar in NCI-H1975-OsiR-PDK1^-/-^ and NCI-H1975-OsiR-PDK1^++/++^ clones, but its phosphorylation level was significantly reduced in PDK1 knock-out cells, and no significant effect on mTOR or pmTOR levels was mediated by osimertinib (Fig. 7b). Osimertinib treatment was able to inhibit the phosphorylation of EGFR (Tyr1173) completely in parental cells (NCI-H1975), although total EGFR level was increased in sensitive and remained unaffected in resistant cells by osimertinib (Suppl Fig. 1). A very low level of EGFR was found in PDK1 knockout in NCI-H1975-OsiR cells, which was significantly increased when PDK1 was rescued in PDK1 KO cells (NCI-H1975-OsiR-PDK1^++/++^ Suppl Fig. 8A). PTEN is a tumor suppressor that regulates the PI3K/AKT/mTOR pathway important in senescence and apoptosis^31^. Analysis of PTEN total expression showed that the expression of PTEN was downregulated in NCI-H1975-OsiR compared with NCI-H1975 (Fig. 7c). Phosphorylation of PTEN was upregulated by osimertinib treatment only in NCI-H1975. Potential signaling pathway interactions regulated by PDK1 are shown graphically in Suppl Fig. 8B.

### PDK1 knock-out promotes cell cycle arrest at G1

Cell cycle analysis showed that osimertinib promoted cell cycle arrest at G1 in NCI-H1975-sensitive cells and significantly reduced the number of cells in S and G2 phases (Fig. 7d, left; E). In contrast, a very low number of cells was found in G1 phase, which was not altered by osimertinib treatment in NCI-H1975-OsiR cells (Fig. 7d, middle; E). A large number of NCI-H1975-OsiR cells accumulated at G2 and G2/M that remained statistically unaffected by osimertinib treatment (Fig. 7d, middle; E). Osimertinib in PDK1 knock out also promoted cell cycle arrest at the G1 phase, to the same extent as in NCI-H1975-sensitive cells (Fig. 7d, middle; E). NCI-H1975-OsiR-PDK1^++/++^ cells were not arrested at G1 post osimertinib treatment, which is similar to NCI-H1975-OsiR cells (Fig. 7d, right; E). These results suggest that PDK1 knock-out renders the resistant cells more sensitive to osimertinib through cell cycle arrest at G1.

### Upregulation of YAP and PDK1 knock-out inhibits YAP expression and nuclear translocation

**Fig. 8:**
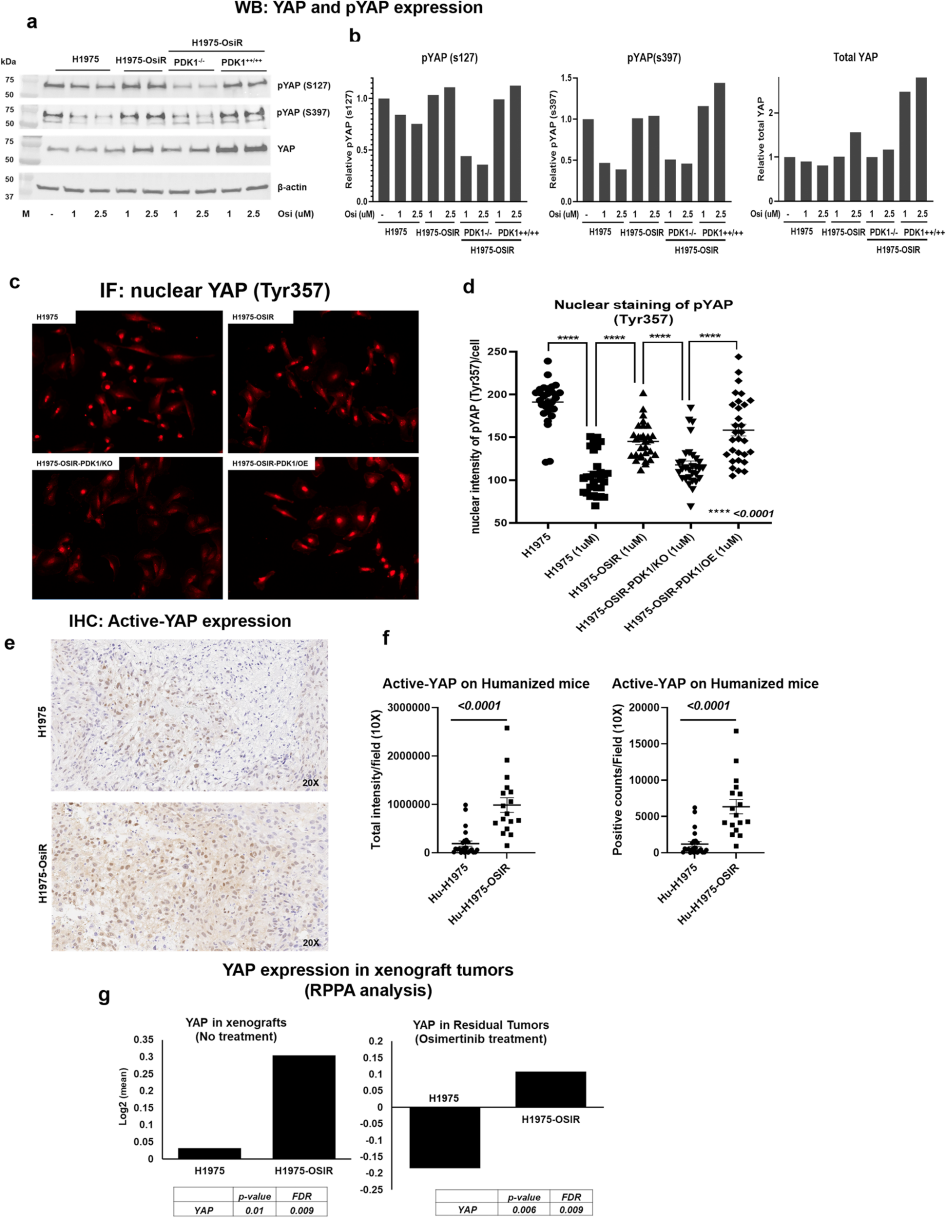
PDK1 knock-out inhibits YAP expression and nuclear translocation. **a** Western blot shows expression of total YAP, pYAP (S127), pYAP (S397) in osimertinib-sensitive NCI-H1975, resistant NCI-H1975-OsiR, NCI-H1975-OsiR-PDK1^-/-^ and NCI-H1975-OsiR-PDK1^++/++^ cells, **b** Quantitation of pYAP (S127), pYAP (S397) and total YAP in NCI-H1975 isogenic cell lines, **c** Immunoflourescence images of nuclear translocation of YAP detected by immunostaining with pYAP (Tyr 357) antibody on osimertinib-sensitive and resistant cell lines as well as PDK1 knockout and re-expressing cells, **d** Quantitative analysis of nuclear YAP signals in four NCI-H1975 isogenic cell lines, **e** Active-YAP expression was evaluated by IHC using anti-active-YAP antibody on NCI-H1975 and NCI-H1975-OsiR xenograft tumors. **f** Active-YAP signals were quantitated from IHC sections by imageScopre using 15–20 10X images per group. **g** Level of YAP was determined from RPPA analysis of osimertinib-sensitive and resistant xenograft tumors (left panel) and osimertinib treated residual sensitive and resistant tumors (right panel).

Increased expression of YAP was found in osimertinib-resistant NCI-H1975-OsiR cells compared to parental cells. Osimertinib treatment reduced the level of YAP in sensitive cells (NCI-H1975) in a dose-dependent manner, whereas YAP expression increased in NCI-H1975-OsiR cells after osimertinib treatment (Fig. 8a). A significantly increased level of total YAP was found in NCI-H1975-OsiR xenograft tumors as compared with the parental NCI-H1975 tumor (Fig. 8g, left). Osimertinib treatment downregulated YAP expression in parental tumors, but significantly upregulated in NCI-H1975-OsiR tumors (Fig. 8g, right). Phosphorylation of YAP at S127 and S397 sites, which are inhibitory for YAP were significantly downregulated by osimertinib treatment in sensitive cells but unchanged in NCI-H1975-OsiR cells (Fig. 8a, b). We also determined the YAP and pYAP status in PDK1 knockout cells. Knockout of PDK1 in osimertinib-resistant cells (NCI-H1975-OsiR-PDK1^-/-^) downregulated YAP and pYAP (s127 & s397) whereas NCI-H1975-OsiR-PDK1^++/++^ had increased levels of YAP and pYAP expression (Fig. 8a, b). Overall, NCI-H1975-OsiR and NCI-H1975-OsiR-PDK1^++/++^ had higher levels of pYAP than NCI-H1975-OsiR-PDK1^-/-.^ Phosphorylation of YAP at Y357 is activating and promotes translocation of YAP to the nucleus. An anti-pYAP^Y357^ antibody was used to localize YAP^Y357^ by immunofluorescence. In osimertinib-sensitive cells (NCI-H1975), osimertinib treatment significantly reduced nuclear localization of pYAP^Y357^ (Fig. 8d). NCI-H1975-OsiR and NCI-H1975-OsiR-PDK1^++/++^ cells showed a high level of nuclear localization of pYAP^Y357^, whereas PDK1 knockout significantly downregulated the nuclear localization of pYAP^Y357^ in NCI-H1975-OsiR-PDK1^-/-^ cells (Fig. 8c, d). We also verified the status of active-YAP in osimertinib-resistant xenograft tumors by IHC using a YAP antibody, which selectively detects nuclear YAP. The IHC results showed that a high level of active-YAP was found in NCI-H1975-OsiR xenograft tumors, which is statistically significant as compared with the level found in NCI-H1975 tumors (Fig. 8e, f).

### Immunohistochemical analysis shows high PDK1 expression in EGFR mutant NSCLC patients with progressive disease

Formalin-fixed paraffin-embedded (FFPE) tumor blocks from EGFR mutant NSCLC tumors obtained prior to initiation of treatment or after tumor progression during treatment were stained with anti-PDK1 antibody for IHC analysis. The highest levels of PDK1 expression were only observed in the progressive disease patients, suggesting they could be responsive to an osimertinib and PDK1 inhibitor combination (Supplemental Fig. 9). To see the association between PDK1 and YAP expression in clinical samples, progressive disease samples were stained with both PDK1 and YAP. The samples with PDK1 score 2+ or more have a higher percentage of nuclear YAP staining (Suppl Fig. 10).

## Discussion

Responses to osimertinib and other TKIs are transient, and acquired resistance is inevitable. The majority of EGFR mutant NSCLC patients treated initially with osimertinib will eventually progress after only 19 months of treatment^37^. Although acquired new mutations in EGFR account for some clinical acquired resistance to both osimertinib and other TKIs, the majority of resistant phenotypes cannot be explained by acquisition of these mutations. Delaying and treating tumors with acquired resistance requires an understanding of multiple complex resistance mechanisms mediated by alternative bypass pathways. In this study, we used the extensively molecularly characterized human NSCLC NCI-H1975 cell line which harbors two common EGFR point mutations, T790M and L858R, in exons 20 and 21, respectively, which is very sensitive to osimertinib, and its isogenic derivative osimertinib-resistant clone as a model system, to first develop a relevant humanized mouse model that models osimertinib acquired resistance accurately, and second, decipher cellular and molecular mechanisms for its acquired resistance. The NCI-H1975-resistant clone, NCI-H1975-OsiR, is 100–200-fold more resistant to osimertinib. Whole-exome sequencing eliminated the acquisition of new EGFR mutations or loss of T790M and L858R in the resistant clone, suggesting the existence of alternative mechanisms of resistance acquired during osimertinib treatment.

Like in vitro resistance, the NCI-H1975-OsiR xenograft tumors showed in vivo resistance in NSG mice. The parental NCI-H1975 tumors were very sensitive to osimertinib with long and durable response whereas the resistant tumor showed significantly less responsive to the drug. A set of immune-related protein expressions such as STING, MIF, CD20, B7-H3, HMHA1, HLA-DQA1, Granzyme-B, B7-H4, PD-L1 were altered in resistant tumors or tumors after prolong osimertinib treatment. A set of metabolic proteins including glutaminase, Pdcd4, glutamate, PDHA1, GCLC, FGF-basic were also significantly upregulated after continuous treatment of osimertinib. When compared to current mouse models, our humanized mouse model replicates human EGFR mutant tumor growth physiology, pathology, immunology, and response to osimertinib treatment. In our recent published report, we showed that HLA matching between CD34 stem-cell donors and inoculated tumors or implanted PDXs, was not necessary for an antigen-specific antitumor response^35^. Using this model, we investigated responses to osimertinib on both NCI-H1975 and NCI-H1975-OsiR tumors developed in fully competent humanized mice. Similar to the NSG mice, the resistant tumors were significantly less sensitive to osimertinib as compared with parental tumors. Osimertinib-sensitive and resistant humanized xenografts had different responses to short and long-term treatment, with the latter having initially a slowing of growth followed by aggressive tumor regrowth. Sensitive tumors had very long regression, before tumors started to regrow slowly similar to responses seen in the clinic. Although the resistant tumor growth was not rapid in mice with the growth rate slower than the parental tumor, resistant tumors did not regress under constant osimertinib pressure, which is consistent with the osimertinib-resistant TC386 PDX growth pattern and clinical observations of slow progression following osimertinib acquired resistance.

Pairwise comparison analysis of protein expression profiles of residual tumors in osimertinib treated NSG mice, using reverse-phase protein array (RPPA), between NCI-H1975 compared to NCI-H1975-OsiR, and NCI-H1975-OsiR osimertinib treated vs. untreated groups, showed two distinct protein signatures. We found unexpectedly that both signatures were led by upregulation of PDK1, a master regulator the AGC kinase superfamily^22^. PDK1 is also upregulated after prolonged osimertinib treatment in NCI-H1975 cells. PDK1 expression detected by IHC was increased significantly in osimertinib-resistant tumors developed in humanized and non-humanized mice. PDK1 regulates the oncogenic PI3k/AKT/mTOR pathway, which is involved in tumorigenesis and progression of NSCLC. Global and phospho-proteome-based mass spectrometry (MS Spec) analyses between NCI-H1975 and NCI-H1975-OsiR clones did not find any detectable increase in PDK1 expression level but indicated a highly significant 14-fold increase of phosphorylated PDK1 in the resistant clone. A study by Zhang et al analyzing an osimertinib-resistant cell line with mass spectrometry did not find PDK1 significantly upregulated protein but found considerable differences in protein expression between moderately vs. heavily resistant isogenic cell lines^36^. This is most probably due to low levels of basal expression of PDK1 in cell lines as compared to xenograft tumors and PDXs. In our study, a very high level of both PDK1 and pPDK1 expression was found in TC386-OsiR PDXs compared to the NCI-H1975-OsiR cell line. Consistent with the Zhang et al study, our study also found enrichment of the PI3K/AKT/mTOR signaling pathways as a mechanism of resistance. Pharmacological and genetic suppression of PDK1 sensitized NCI-H1975-OsiR cells to osimertinib. Cell survival and colony formation assays showed that NCI-H1975-OsiR clones treated with the specific PDK1 inhibitor, BX795, or PDK1 knock out by CRISPR/Cas9, recovered their sensitivity to osimertinib treatment. In addition, NCI-H1975-OsiR PDK1 knockout clone NCI-H1975-OsiR-PDK1^-/-^, with restored overexpression of PDK1 was more resistant to osimertinib than its parental clone, validating the role of PDK1 in mediating osimertinib resistance in this model. Treatment with a combination of osimertinib and BX795 in a second model of acquired osimertinib resistance utilizing a PDX showed that the addition of BX795 to osimertinib resulted in synergistic tumor regression whereas BX795 treatment did not differ from untreated control growth and osimertinib slowed growth but did not cause regression in the drug-resistant PDX. This PDX model with acquired osimertinib resistance without a T790M mutation replicates a common clinical scenario that suggests a combination of osimertinib with a PDK1 inhibitor may be effective after progression on first-line osimertinib.

PDK1 is a pivotal node in the oncogenic PI3K/AKT/mTOR pathway^20,21^, which mediates tumorigenesis and resistance to EGFR tyrosine kinase inhibitors in NSCLC^16–19^. Osimertinib had no effect on AKT expression levels, which was similar in both PDK1 knock out and overexpressing clones. In the latter, osimertinib reduced AKT phosphorylation at the threonine 308 (T308) residue, which is known to be the site activated by PDK1. The level of mTOR expression was also similar in PDK1 knock out and overexpressing clones, but its phosphorylation level was higher in the latter indicating that PDK1 knock out can downregulate mTOR activation. A previous study by our group implicated mTOR as a mediator of TKI resistance^38^. In this study three NSCLC cell lines became sensitive to erlotinib following treatment with the mTOR inhibitor rapamycin.

PDK1 is also a mediator of yes-associated protein (YAP) activation^39^. PI3K and PDK1 mediate YAP phosphorylation and nuclear accumulation, and thus it is the PI3K-PDK1 signal that links EGFR with the Hippo pathway. Phosphorylation of YAP at Y357 is activating, resulting in YAP nuclear translocation^40^. Osimertinib treatment significantly inhibited YAP translocation by inhibiting the phosphorylation of YAP Y357 in sensitive cells (NCI-H1975), which is increased in NCI-H1975-OsiR cells. PDK1 knockout NCI-H1975-OsiR-PDK1^-/-^ cells significantly decreased YAP Y357 nuclear translocation whereas rescuing PDK1 into PDK1 knockout cells, NCI-H1975-OsiR-PDK1^++/++^, reverted YAP nuclear translocation, implicating YAP as a downstream mediator of osimertinib acquired drug resistance. A significantly higher level of active-YAP expression was found in NCI-H1975-OsiR tumors in humanized mice, which was detected by IHC using an antibody that only detects nuclear YAP. This is supported by studies implicating YAP activation in persister cells after EGFR TKI treatment^41^. We recently reported YAP-driven transcriptional adaptation as a functional mechanism of TKI drug tolerance^42^. In this study, we found in experiments in humanized mice that YAP reduced treatment sensitivity to osimertinib and enhanced an immunosuppressive tumor microenvironment supporting tumor growth. Thus, PDK1 is a central upstream regulator of two critical drug resistance pathways: PI3K/AKT/mTOR and YAP. This suggests that drugs targeting PDK1 could be beneficial in delaying the onset of acquired drug resistance and treating acquired drug resistance at its onset.

Cell cycle analysis showed that both osimertinib treatment of sensitive cells and PDK1 knockout promoted cell cycle arrest at the G1 phase, whereas resistant and PDK1 re-expressors in PDK1 KO cells were not arrested at G1. Osimertinib-resistant cells, NCI-H1975-OsiR, showed a higher level of a 2nd peak that may be from subpopulations of different ploidies and may be considered as a second G1 peak. However, regardless of the number of peaks, osimertinib did not arrest cells in G1 in this Osimertinib-resistant population. PDK1 is known to have a critical role in cell proliferation and cell cycle progression^43^. Finally, we showed that high expression of PDK1 was associated with progressive disease in EGFR mutant NSCLC patients, as shown by immunohistochemistry (IHC), suggesting they could be responsive to osimertinib and PDK1 inhibitor combination therapy.

In conclusion, we presented multiple lines of evidence for PDK1 as a driver of osimertinib acquired resistance in T790M/L858R mutant NSCLC using the most relevant preclinical mouse models capable of modeling osimertinib acquired resistance We showed that pharmacological and genetic targeting of PDK1 could restore osimertinib responsiveness in cell lines and PDXs with acquired osimertinib resistance thus providing support for clinical translation.

## Methods

### Osimertinib-resistant cells, cell culture, and maintenance

The human parental NCI-H1975 NSCLC cell line, which carries EGFR T790M/L858R mutations, and its osimertinib-resistant isogenic clone, NCI-H1975-OsiR, were obtained from Dr. John Minna’s laboratory (University of Texas Southwestern University, Dallas, TX), and Dr. John Heymach’s laboratory (University of Texas MD Anderson Cancer Center (MDACC), Houston, TX), respectively. NCI-H1975-OsiR cells were cultured and maintained in RPMI-1640 complete media supplemented with 10% heat-inactivated fetal bovine serum (GE Healthcare Life Sciences, HyClone Laboratories) and 1% penicillin–streptomycin (Thermo Fisher Scientific) and 1 µM osimertinib (Medchemexpress (MCE), NJ, USA), which was dissolved in DMSO, stored at −70 °C, and diluted in culture medium for in vitro experiments. There have been no publications on this resistant cell line.

### Antibodies

Antibodies were purchased from Cell Signaling (Beverly, MA): anti- AKT (CS#4691), p-AKT (Ser473) (CS#9271), p-AKT (Thr308) (CS #4056), mTOR, p-mTOR (Ser2448) (CS#2971), PDK1 (CS#13037), p-PDK1(Tyr373/376) (bs-3017R), p-PDK1(Ser241) (CS#3061), MAPK (CS#4695), p-MAPK (Thr202/Tyr204) (CS#4370), PTEN (CS#9559), p-PTEN (CS#9549), YAP (CS#4912), p-YAP (s127) (CS#13008), p-YAP (s397) (CS#13619). Monoclonal anti-β-actin (Sigma#A5449) was purchased from Sigma-Aldrich (St Louis, MO).

### Generation of PDK1-knockout and PDK1 overexpression cells

PDK1-knockout clones (NCI-H1975-OsiR-PDK1^-/-^) were generated with CRISPR-Cas9 technology (CRISPR core lab, Baylor College of Medicine (BCM), Houston, TX). The PDK1 re-expressing clone was generated by the BCM core lab, by stable transfection with Myc-tagged PDK1 overexpressing plasmid into NCI-H1975-OsiR-PDK1^-/-^ clone (OriGene, Rockville, MD).

### Whole-exome sequence analysis

NCI-H1975 and NCI-H1975-OsiR cells were seeded in triplicates at a cell density of 2 × 10^6^/plate. DNA was isolated and purified using a Qiagen kit (Germantown, MD). The quality of DNA was evaluated and the whole-exome sequenced at the sequencing core lab (MDACC, Houston, TX) using a next generation sequencer (NextSeq500, Illumina, USA). Sequencing data were analyzed by the Department of Bioinformatics and Computational Biology, MDACC.

### Drug sensitivity assay

NCI-H1975 and NCI-H1975-OsiR isogeneic cells were seeded at a density of 3×10^3^ cells/well in a 96-well microplate and treated with osimertinib at concentrations ranging from 0.001 to 10 µM in DMSO. Cells were incubated in 37 °C incubators, 5% CO_2_, for three days. Cytotoxicity assays were performed using colorimetric XTT (Sigma-Aldrich, USA) and SRB (Sulforhodamine B) Assay Kit (Abcam, USA) reagents according to manufactured protocol. Optical density (OD) was measured using a microplate reader (FLUOstar Omega, BMG Labtech USA) at 570 nm. Cell viability (%) = [OD (Drug) – OD (Blank)]/[OD (Control) - OD (Blank)] × 100.

### Development of osimertinib-sensitive and resistant tumor xenografts in humanized mice

NOD.Cg-*Prkdc*^*scid*^ *Il2rg*^*tm1Wjl*^/SzJ (NSG) mice were obtained from The Jackson Laboratory. All animal experiments were carried out following approval by the MDACC institutional review board and were performed in accordance with the Guidelines for the Care and Use of Laboratory Animals published by the National Institutes of Health. All measurements quantifying experimental outcomes were blinded to the intervention. For generation of humanized mice, human umbilical cord blood units were obtained from MD Anderson Cord Blood Bank under an Institutional Review Board (IRB)-approved protocol (Lab04-0249). The cord blood bank collects umbilical cord blood through voluntary donations from mothers following informed consent under the institutional approved IRB protocol. After mononuclear cells were separated from human umbilical cord blood, CD34^+^ HSPCs were isolated using a CD34^+^ MicroBead kit (Miltenyi Biotec). Three- to 4-week-old NSG mice were irradiated with 200 cGy using a ^137^Cs gamma irradiator. Over 90% pure freshly isolated HLA typed CD34^+^ HSPCs were injected intravenously, twenty-four hours post irradiation, at a density of 1 to 2 × 10^5^ CD34^+^ cells/mouse. Ten mice per group from multiple umbilical cord blood donors were used. The engraftment levels of human CD45^+^ cells were determined in the peripheral blood, as early as 4 weeks post CD34 injection, by flow cytometric quantification, as well as other human immune populations. Mice with 25% human CD45^+^ cells were considered as humanized (Hu-NSG mice). The reconstitution levels of human immune cell populations in mice was analyzed throughout experiments using a 10-color flow cytometry panel at week 6 post CD34^+^ engraftment. These include CD45^+^, CD3^+^, CD4^+^, and CD8^+^ T cells, B cells, NK cells, dendritic cells (DC), myeloid derived suppressor cells (MDSC), and macrophages. Hu-NSG mice from different cord blood donors with different levels of engraftment were randomized into every treatment group in every experiment. All Hu-NSG mice were verified for humanization before tumor implantation. Treatment strategies for different experiments are described in the figures. To maintain the resistance potentials, NCI-H1975-OsiR xenograft tumors were grown under continuous oisimertinib pressure by treating mice one day after cell injection.

### Generation of PDXs with acquired resistance to osimertinib

To develop NSCLC PDXs with acquired resistance for osimertinib, we monitored mice with regressed tumors for tumor regrowth. When those tumors regrew to 200 mm^3^ in size, we retreated, until mice were euthanized. The tumors were passaged to new NSG mice for osimertinib sensitivity testing. PDX TC386, is in passage 4, with each generation treated with three or more cycles. Susceptibility to osimertinib was reduced in each passage. We performed whole-exome sequencing for two tumors obtained in passage 3 (G3) of TC386 that was under constant osimertinib treatment and did not regress during osimertinib treatment. Both G3R1 and G3R2 had the same EGFR exon 19 deletion as the primary tumor (TC386T) and parental PDX (TC386F2), albeit with increased allele frequencies without novel EGFR mutations including the absence of T790M. The PDXs with acquired resistance had new mutations that were not detected in either the primary tumor or parental PDX, including mutations in FAT3 and SETD1B.

### Immune profile analysis by flow cytometry

Harvested fresh tumors were processed for single-cell suspensions by enzymatic digestion (Liberase Enzyme Blend, Roche, USA). Erythrocytes in the peripheral blood were lysed with ACK lysis buffer (Fisher Scientific). Several 10-color flow cytometry panels were used for immune profiling of innate and adaptive immune populations. Fluorochrome–conjugated monoclonal antibodies to the following human antigens were used: CD45-Alexa Fluor 700 (clone 2D1, HI30), CD45-phycoerythrin (PE; clone 2D1, HI30), CD3-PerCp/cy5.5 (clone HIT3a), CD19-PE-cyanine 7 (clone HIB19), CD56-PE/BV510 (clone HCD56). A mouse CD45-FITC (clone 30-F11) antibody was used for gating out murine leukocytes. Antibodies were purchased from Biolegend. The dilutions of antibodies used in sample staining were followed according to the manufacturer protocol. All samples were run on Attune NxT flow cytometer (Thermo fisher), and data were analyzed by Flow Jo software.

### Development of osimertinib-sensitive and resistant tumor xenografts in non-humanized mice

NCI-H1975-OsiR isogenic cells were cultured and expanded in osimertinib (1 µM) containing media. For sensitive xenograft, NCI-H1975 at 5 × 10^6^ cell density were injected subcutaneously into 6–8-week-old NSG mice. When tumor size reached approximately 100 mm^3^, tumor-bearing mice were randomized and treated with osimertinib, 5 mg/kg or 10 mg/kg, 5 days a week for 3 weeks. To develop resistant xenograft, NCI-H1975-OsiR cells (5 × 10^6^) were injected subcutaneously into 6–8-week-old NSG mice followed by osimertinib treatment starting from the day following tumor cell injection so tumors developed under osimertinib pressure. Mice were treated with designated concentration of osimertinib from the first day of tumor cell injection throughout the entire experiment, and tumor sizes were measured twice a week by caliper. To evaluate the effect of the PDK1-selective inhibitor, BX795, 5 tumor-bearing mice/group were either left untreated, treated with osimertinib alone (5 mg/kg), treated with PDK1 inhibitor BX 795 (25 mg/kg) (Selleckchem, Houston, TX), or with the combination. Tumor volume was measured using the formula *V* = *ab*^2^/2 where a is the largest diameter and b is the smallest. At end of the experiment, residual tumor tissues were harvested for further analysis.

### In vivo inhibition of PDK1 in an osimertinib-resistant NSCLC PDX

To evaluate the effect of PDKi, BX 795, we propagated the EGFR mutant TC386-OsiR PDX with acquired osimertinib resistance in NSG mice. After 3 weeks, fresh PDXs were harvested and 2 × 2 cm size PDX tissues were re-implanted into 25 NSG mice for the experiment. Large size PDX bearing mice were randomized into four groups including control, osimertinib alone (10 mg/kg), BX 795 alone (25 mg/kg) and an osimertinib + BX 795 combination group. Osimertinib treatment was 5 days a week (oral) and BX 795 treatment was 2 times per week (i.p.). Tumor size was measured twice a week by caliper. Tumor volume was calculated according to the formula *V* = *ab*^2^/2, where a is the largest diameter and b is the smallest. At the end of the experiment, residual tumor tissues were harvested for analysis.

### Western blot analysis

Total protein was harvested using Ripa lysis buffer (Merck, Burlington, MA), and their concentrations were evaluated with BCA^TM^ protein assay kit (Pierce, Rockford, IL, USA). Equal amounts of proteins were separated by 8–15% SDS-PAGE gel, electro-transferred onto a Hybond ECL transfer membrane (Amersham Pharmacia, Piscataway, NJ), and blocked with 2–5% non-fat skim milk. Then, membranes were probed with specific primary antibodies at 1:1000 dilution for overnight at 4 °C, washed with PBS, and incubated with corresponding secondary antibodies at 1:2000 dilution at room temperature for 1 h. The specific protein bands were visualized with an ECL advanced western blot analysis detection kit (GE Health Care Bio-Sciences, NJ, USA).

### Colony formation assay

NCI-H1975, NCI-H1975-OsiR, NCI-H1975-OsiR-PDK1^-/-^ and NCI-H1975-OsiR-PDK1^++/++^ cells were seeded into 6-well plates at a density of 300 cells per well, and treated with 0, 10 nM, 100 nM, 1 µM, 2.5 µM, and 5 µM concentrations of osimertinib for 72 h. The media was replaced every 2–3 days with osimertinib dose titration containing medium. After twelve days, colonies were fixed with 4% PFA, stained with crystal violet solution, and photographed.

### Cell cycle assay

The cell cycle profiles of osimertinib-resistant and sensitive cells were determined by staining DNA with fluorescent dye (PI/RNase staining buffer, BD Pharmingen, USA) according to the manufacturer’s protocol, and measuring its intensity by flow cytometry (Attune NxT, Thermofisher Scientific, USA). Briefly, cells were seeded at 10^6^ cells in a 100 mm dish followed by osimertinib treatment at the designated concentrations. Cell pellets were suspended in ice cold PBS, fixed with 70–80% ethanol, and stored at −20 °C overnight. The cells were washed twice with ice cold PBS and stained with PI/RNase staining dye for 15 min at room temperature. The samples were analyzed by flow cytometry within an hour.

### Reverse-phase protein array (RPPA) analysis

Osimertinib-sensitive and resistant H1975 tumors were developed in non-humanized NSG mice and treated with osimertinib under the protocol described above. Residual tumor tissues were snap-frozen and stored in −80 °C. RPPA analysis was performed using 400 antibodies at the RPPA core lab at MDACC. Bioinformatics analysis was performed by the Department of Bioinformatics and Computational Biology (MDACC).

### Mass spectrometry

NCI-H1975 parental, NCI-H1975-OsiR, NCI-H1975-OsiR-PDK1^-/-^ and NCI-H1975-OsiR-PDK1^++/++^ cell lines were submited for mass spec analysis. All cell lines were maintained in 1 µM osimertinib containing media except NCI-H1975-parental cells. All cells were seeded for 24 h with or without osimertinib treatment (1 µM). Three biological replicates were used in each cell line. The samples were denatured and lysed by three cycles of LN2 snap freeze and thaw at 95 ^o^C. For global profiling, 10 mg of lysate was trypsinized to obtain 10 mg of digested peptides. After fractionation using a small-scale basic pH reverse-phase (sBPRP) step elution protocol with increasing acetonitrile concentrations, fractions were combined into 5 pools that were resolved and sequenced online with Fusion Lumos and timsTOF fleX mass spectrometer. For phospho-proteome profiling, a 100 µg protein lysate was digested with trypsin and dried under vacuum. Global and phospho-proteomic analyses covered over 8000 gene protein products (GPS), and over 4000 GPS, respectively, which included the kinome profile. After label-free nanoscale liquid chromatography coupled to tandem mass spectrometry (nanoLC-MS/MS) analysis using a Thermo Fusion Mass spectrometer, the data was processed and quantified against NCBI RefSeq protein databases in Proteome Discover 2.5 interface with a Mascot search engine (Saltzman, Ruprecht). The Skyline program was used to obtain precise quantification. To decipher phospho-proteome signal pathway analysis we utilized protein external data contributions for phosphorylation-related data mining sets, including PhosphoSitePlus (http://www.phosphosite.org/), Phospho.ELM, PhosphoPep, and the Phosphorylation Site Database (PHOSIDA).

### Immunofluorescence

Cells were seeded at 5000 cells/chamber well and grown overnight before being treated with osimertinib at 1 µM or 2.5 µM for 24 h. Then cells were washed with PBS and fixed in 4% paraformaldehyde in PBS pH 7.4 for 10 min at RT. After washing with PBS 3 times, cells were treated with 0.125% Triton-X100 for 10 min at RT to increase cell permeability. The slides were blocked by 1%BSA block in PBS-T (Thermo-Fisher) at RT for 30 min, and incubated with 1:250 anti-pYAP Y357 (Sigma #Y4646) antibody in 1%BSA overnight at 4 °C. After three PBS-T washes, the slides were further incubated in 1:1000 secondary Alexa 594 antibody (Invitrogen #A32741) at RT for 1 h, before they were mounted with mounting media containing DAPI (abcam #104139). Immune fluorescence images were captured using an EVOS M5000 fluorescence microscope (Thermos-Fisher). For each cell line and each treatment condition, 30 individual cell nuclei were counted and their fluorescent intensities were quantified.

### Immunohistochemistry

Formalin-fixed NCI-H1975, NCI-H1975-OsiR xenografts tumors developed in humanized and non-humanized NSG mice were underwent IHC for PDK1 and active-YAP expression. All samples were paraffin embedded, and cut and immune stained in the immunohistochemistry core lab (MDACC). The antibodies for PDK1 (clone EP569Y, # ab52893), and active-YAP (EPR19812, #ab205270) were purchased from Abcam. Antibodies were validated in the core lab before performing IHC. Clinical specimens of NSCLC samples were obtained from patients before initiating systemic targeted therapy (TKI naive [TN]), at the residual disease (RD) state, and upon subsequent progressive disease as determined by clinical imaging, at which point the tumors showed acquired drug resistance (progression [PD]). All patients gave informed consent for collection of clinical correlates, tissue collection, and research testing under Institutional Review Board (IRB)-approved protocols. Patient studies were conducted according to the Declaration of Helsinki, the Belmont Report, and the U.S. Common Rule. Formalin-fixed paraffin-embedded (FFPE) tumor blocks were cut at 4-micron thickness and mounted as sections on positively charged histology slides. Immunohistochemistry staining was performed as described previously. Slides were deparaffinized in xylene, rehydrated and epitope retrieval was induced in a histology pressure cooker using pH 6.1 citrate buffer (Dako Denmark A/S, S2369). After endogenous peroxidase, tissue was permeabilized in in 0.1% Triton-X/PBS. Non-specific binding was blocked, and slides were incubated primary antibody solution overnight at 4 °C. The antibody for PDK1 (clone EP569Y, # ab52893) was purchased from Abcam and diluted 1:150. Then, slides were incubated with secondary antibody for 30 minutes (EnVision Dual Link Labeled Polymer HRP, Agilent K4065), stained using 3,3-DAB, and counterstained with hematoxylin. Slides were dehydrated and mounted before digitization using an Aperio AT2 Slide Scanner (Leica Biosystems) at a 20X objective.

### Statistics and reproducibility

#### WES

The quality of raw FASTQ reads was assessed using FastQC and then mapped to human reference genome GRCh38, using BWA^44,45^. The reference genome refers to the b38 version with decoy sequences for human GRCh38 provided in the genome analysis toolkit (GATK) resource bundle^46^. The mutations were called following GATK best practice pipeline. The candidate mutations were be filtered for high confident somatic mutations and annotated for functional changes using ANNOVAR^47^. At least *N* = 3 samples/group were analyzed for WES.

#### Cell survival assay

The percentage of viable cells was determined by the ratio of absorbance of treatment and control groups: ODT/ODC x 100%. Univariate analysis was performed to evaluate the distribution of data for each treatment group. To determine whether SRB % was different between treatment groups, two methods were used: (1) ANOVA was performed to compare the variance between treatment groups for all samples within each cell line; and (2) Tukey’s multiple comparisons test was performed for pairwise differences between treatment groups. *P* < 0.05 was considered statistically significant; all tests were two-sided. Analyses were performed using SAS 9.3 (SAS Institute Inc., Cary, NC). Values represent the mean of three independent experiments. Each assay was performed *N* ≥ 3 times with at least 3 replicates in each time.

#### Colony formation assay

Colonies were fixed with glutaraldehyde, stained with crystal violet, counted with a stereomicroscope, and analyzed with Image-J software. Values represent the mean of three independent experiments. The statistical significance of differences between treatment groups was calculated by two-tailed *t*-test analysis; *P* < 0.05 was considered significant. The Statistical software S-PLUS 8.0 was used for all analyses. Each assay was performed *N* ≥ 3 times with at least 3 replicates in each time.

#### Tumor growth

Statistical analyses were performed with GraphPad Prism 7 software. Tumor intensity change per time point was calculated as a relative level of tumor intensity change from baseline. Two-way ANOVA with interaction of treatment group and time point was performed to compare the difference of tumor intensity changes from baseline between each pair of the treatment groups at each time point. Means ± standard errors of the mean are shown in all graphs. The nonparametric Mann-Whitney U test was applied to compare cell numbers in different treatment groups. Differences of *P* < 0.05, *P* < 0.01, and *P* < 0.001 were considered statistically significant. Statistical analysis of flow cytometry data was done by general linear regression models to compare the data among the different treatment groups. CONTRAST statement in PROC GENMOD procedure in SAS was used to compare the data between each pair of the treatment groups with treatment indicator in the models. Both nom*P*-values and multiple testing adjusted *P*-values were reported. SAS version 9.2 and S-Plus version 8.04 were used for the computations for all analyses. All in vivo tumor growth and treatment experiments were repeated *N* = 3 times with *N* = 5–10 mice/group in each time.

#### Reverse-phase protein array (RPPA)

Slides were scanned using a CanoScan 9000F and spot intensities were quantified using ArrayPro Analyzer 6.3 (Media Cybernetics Washington DC). SuperCurve, a software developed in house, was used to estimate relative protein levels^48^.

After SuperCurve fitting, protein measurements were normalized for loading using median centering across antibodies. One-way analysis of variance (ANOVA) was used to assess the differences in protein expressions between control and treatment groups on a protein-by-protein basis. First, for one feature (protein) at a time, we carried out an over-all F-test to detect any significant difference among the means of all the groups. Next, for the featured (proteins) identified in this process, we then compared between desired groups to identify the sources of difference. The R library “multcomp” was used for this purpose. Note that the FC (fold-change) values were calculated as the estimated ratio between the 2 groups in comparison, with the following conventional modification: For the rations >1 (upregulation), FCs were noted as the same as the ratio. For the rations ≤ 1 (down-regulation), FC were noted as the negative inverse of the ratio. Furthermore, to account for multiple testing, we estimated the false discovery rates (FDR) of the overall test of the model using the Benjamini–Hochberg method. A FDR-adjusted *P*-value less than 0.05 was considered as statistically significant unless otherwise mentioned. The criteria of significant protein selection were: 1. Significant in overall *F*-test (FDR-adjusted *P*-value < 0.05); 2. Significant in pairwise comparison (FDR-adjusted *P*-value < 0.05). *N* = 3–5 tumor samples per group were used for RPPA analysis.

#### In vivo inhibition of PDK1 in an osimertinib-resistant PDX

We evaluated the potential synergistic effect of the drug combination osimertinib and BX795 under the Highest Single Agent framework^49^, where the synergistic effect of a drug combination is declared if the combination effect is greater than that of the more effective individual component. The combination index (CI) under the HSA and the corresponding standard error were approximated by the Delta method^50^. We declared the synergistic effect under the significance level of 5% at day 33. The in vivo experiment was repeated *N* = 3 times with at least *N* = 5 mice/group in each time.

#### Mass sectrometry

Statistical analysis was performed using R software (R version 4.0.1). The log2 transformation was applied to the iFOT Half Min proteomic data. The Student’s t-test was used to compare expression values between the groups. *P*-values obtained from multiple tests were adjusted using FDR. Statistical significance was defined as FDR < 0.05. The enriched pathways and hallmarks were identified by pre-ranked GSEA using the gene list ranked by log-transformed *P-*values with signs set to positive/negative for a fold-change of >1 or <1, respectively. *N* = 3 samples/group were analyzed for mass spectrometry.

#### Immunofluorescence and others

We performed multiple *t*-test analysis using GraphPad Prism 9 software. The experiment was repeated at least three times.

### Reporting summary

Further information on research design is available in the Nature Portfolio Reporting Summary linked to this article.

## Supplementary information

Supplementary Infomation

Description of Additional Supplementary Data

Supplementary Data 1

Reporting Summary

## Data Availability

All data generated or analyzed during this study are included in this published article. The source data underlying most graphs and charts used in this manuscript are provided as a excel file in Supplementary Data 1. All uncropped and unedited western blot images used in this manuscript are provided as supplementary information in Supplementary Figure. 12. Request for any source data or materials that are not provided should be made to the corresponding author.
