# Author Correction: 3-Phosphoinositide-dependent kinase 1 drives acquired resistance to osimertinib

**DOI:** 10.1038/s42003-023-04979-9

**Published:** 2023-06-06

**Authors:** Ismail M. Meraz, Mourad Majidi, Bingliang Fang, Feng Meng, Lihui Gao, RuPing Shao, Renduo Song, Feng Li, Yonathan Lissanu, Huiqin Chen, Min Jin Ha, Qi Wang, Jing Wang, Elizabeth Shpall, Sung Yun Jung, Franziska Haderk, Philippe Gui, Jonathan Wesley Riess, Victor Olivas, Trever G. Bivona, Jack A. Roth

**Affiliations:** 1 Department of Thoracic and Cardiovascular Surgery, The University of Texas MD Anderson Cancer Center, Houston, TX USA; 2 Department of Biostatistics, The University of Texas MD Anderson Cancer Center, Houston, TX USA; 3 Department of Biostatistics, Graduate School of Public Health, Yonsei University, Seoul, Korea; 4 Department of Bioinformatics and Computational Biology, The University of Texas MD Anderson Cancer Center, Houston, TX USA; 5 Department of Stem Cell Transplantation, The University of Texas MD Anderson Cancer Center, Houston, TX USA; 6 Department of Biochemistry, Baylor College of Medicine, Houston, TX USA; 7 Department of Medicine, University of California, San Francisco, San Francisco, CA USA; 8 Helen Diller Family Comprehensive Cancer Center, University of California, San Francisco, San Francisco, CA USA; 9 Department of Cellular and Molecular Pharmacology, University of California, San Francisco, San Francisco, CA USA; 10 University of California Davis Comprehensive Cancer Center, Sacramento, CA USA

**Keywords:** Non-small-cell lung cancer, Cancer therapeutic resistance

Correction to: *Communications Biology* 10.1038/s42003-023-04889-w, published online 11 May 2023.

In the original version of the article, the Competing Interests statement read as:

‘J.A.R. reports receiving a commercial research grant from The University of Texas MD Anderson Cancer Center, sponsored research agreement from Genprex, Inc, has ownership interest (including stock, patents, etc.) in Genprex, Inc., and is a consultant/advisory boardmember for Genprex, Inc. T.G.B. is an advisor to Array/Pfizer, Revolution Medicines, Springworks, Jazz Pharmaceuticals, Relay Therapeutics, Rain Therapeutics, Engine Biosciences, and receives research funding from Novartis, Strategia, Kinnate, and Revolution Medicines. J.W.R. is an advisor to Blueprint, Beigene, Daiichi Sankyo, EMD Serono, Janssen, Turning Point, Regeneron, Sanofi Aventis and a consultant to Blueprint, Novartis, Boehringer Ingelheim. He also receives research funding from Merck, Novartis, Spectrum, Revolution Medicine, AstraZeneca.The other authors disclosed no potential conflicts of interest or competing interests.’

This has now been changed to:

‘Jack A. Roth reports receiving a commercial research grant from The University of Texas MD Anderson Cancer Center, sponsored research agreement from Genprex, Inc, has ownership interest (including stock, patents, etc.) in Genprex, Inc., and is a consultant/advisory board member for Genprex, Inc. Trever G Bivona is an advisor to Array/Pfizer, Revolution Medicines, Springworks, Jazz Pharmaceuticals, Relay Therapeutics, Rain Therapeutics, Engine Biosciences, and receives research funding from Novartis, Strategia, Kinnate, and Revolution Medicines. Jonathan Wesley Riess is an advisor to Blueprint, Beigene, Daiichi Sankyo, EMD Serono, Janssen, Turning Point, Regeneron, Sanofi Aventis and a consultant to Blueprint, Novartis, Boehringer Ingelheim. He also receives research funding from Merck, Novartis, Spectrum, Revolution Medicine, AstraZeneca. Elizabeth Shpall is involved in consulting/Scientific advisory board/Speaking in Adaptimmune, Navan, Celaid Therapeutics, Zelluna Immunotherapy, FibroBiologics and Axio. She has also license agreement with Takeda, Affimed and Syena. The other authors disclosed no potential conflicts of interest or competing interests.’

This has been updated in the PDF and HTML versions.

