# Supplementary material for: 3-Phosphoinositide-dependent kinase 1 drives acquired resistance to osimertinib
Source: Commun Biol. 2023 May 11;6:509. doi: 10.1038/s42003-023-04889-w (PMC10175489; doi:10.1038/s42003-023-04889-w)
Supplement: Supplementary file 1 — Supplementary Infomation [file 42003_2023_4889_MOESM1_ESM.pdf]

Supplementary Figures

a

New mutations found in H1975-OsiR cells

Gene	ChangeType
RGSL1	nonsynonymous SNV
ATP6V1C2	nonsynonymous SNV
RFX8	nonsynonymous SNV
TTN	synonymous SNV
TRIM71	synonymous SNV
ABLIM2	nonsynonymous SNV
FYB	nonsynonymous SNV
MOCS1	nonsynonymous SNV
TIAM2	synonymous SNV
TYW1B	nonsynonymous SNV
DGKI	nonsynonymous SNV
LZTS1	synonymous SNV
TEX15	nonsynonymous SNV
SECISBP2	stopgain
COL13A1	nonsynonymous SNV
PKD2L1	synonymous SNV
OR56A3	nonsynonymous SNV
MYOD1	nonsynonymous SNV
PTPRJ	stopgain
OPCML	nonsynonymous SNV
FGF23	nonsynonymous SNV
KRT1	nonsynonymous SNV
CFAP54	nonsynonymous SNV
WSCD2	nonsynonymous SNV
NOS1	nonsynonymous SNV
USPL1	nonsynonymous SNV
MAGEL2	synonymous SNV
DYX1C1	nonsynonymous SNV
SHISA9	nonsynonymous SNV
SMG1	nonsynonymous SNV
TAOK2	nonsynonymous SNV
MYH13	stopgain
L3MBTL4	nonsynonymous SNV
SLC32A1	nonsynonymous SNV
ZBED1	stopgain
MAOB	nonsynonymous SNV
ATP2B3	nonsynonymous SNV

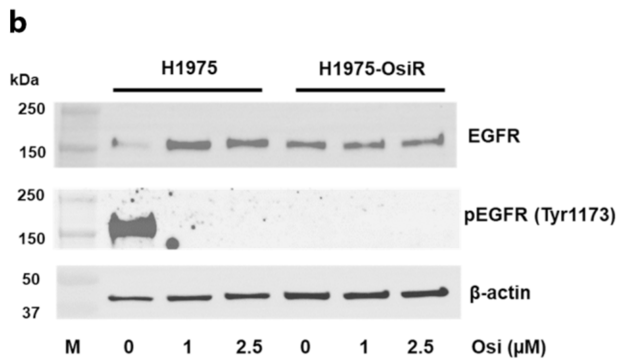

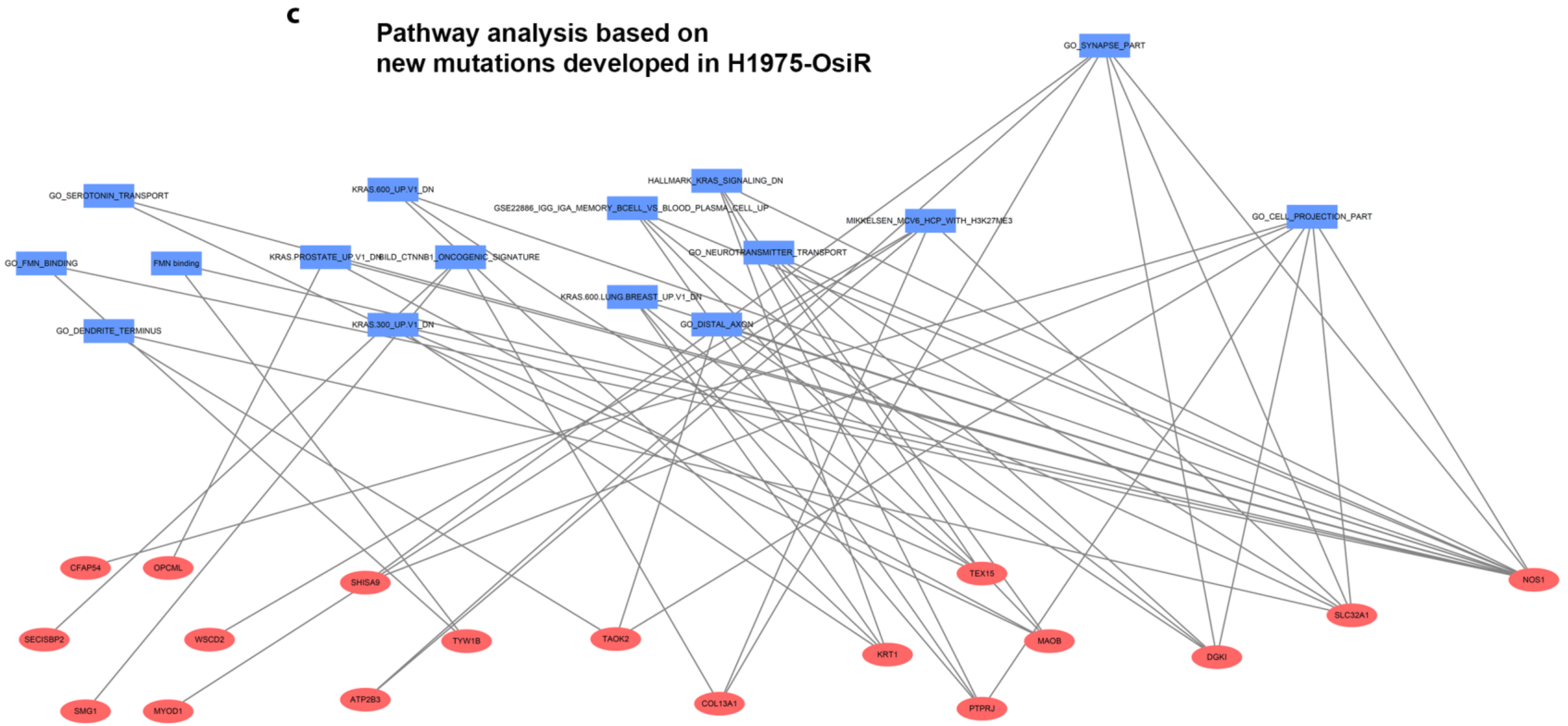

Supplementary Fig 1. Whole exome sequencing shows no new EGFR mutations or loss of the T790M mutation. a) The list of 37 new exonic mutations, among them, 0 indels, 27 nonsynonymous single nucleotide variants (SNV), 4 stopgain and 6 synonymous mutations; **b**) Western blot shows the status of EGFR and pEGFR expression in H1975 and H1975-OsiR cells; **c**) Pathway analysis based on new mutations developed in H1975-OsiR cells.

a

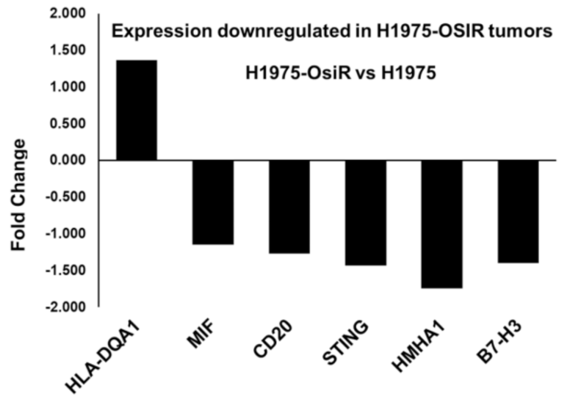

		ANOVA		Pairwise		
		Pvalue	FDR	Diff	Fold-change	Pvalue
HLA-DQA1	Major histocomplatibility complex II	0.000	0.000	0.447	1.363	0.000
MIF	Macrophage inhibitory factor	0.008	0.015	-0.196	-1.145	0.021
CD20	B cell markers	0.000	0.000	-0.345	-1.270	0.000
STING	Involve in antigen presentation	0.001	0.003	-0.517	-1.431	0.000
HMHA1	Minor histocompatibility complex	0.000	0.000	-0.799	-1.740	0.000
B7-H3	Costimulatory molecule for T cells	0.001	0.002	-0.486	-1.401	0.002

b

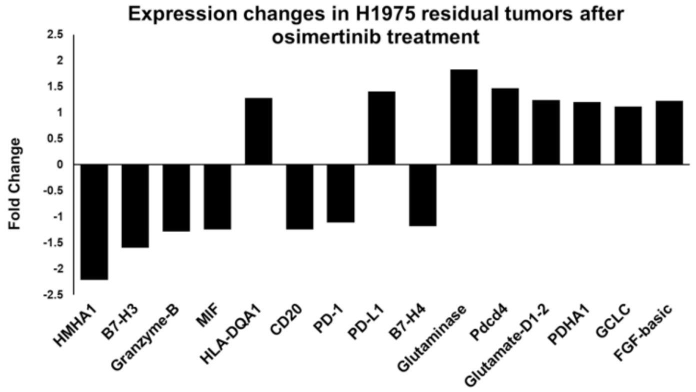

		ANOVA		Pairwise		
		Pvalue	FDR	Diff	Fold Change	Pvalie
HMHA1	Minor histocompatibility complex	5.33E-07	1.25E-05	-1.14379	-2.20961	4.24E-08
B7-H3	Costimulatory molecule for T cells	0.000595	0.001999	-0.67434	-1.59586	0.000129
Granzyme-B		0.001024	0.002946	-0.35749	-1.2812	5.98E-05
MIF	Macrophage inhibitory factor	0.00813	0.014987	-0.31469	-1.24375	0.000818
HLA-DQA1	Major histocomplatibility complex II	1.18E-05	0.000106	0.356496	1.280312	0.000496
CD20	B cell markers	3.30E-06	3.78E-05	-0.31101	-1.24058	0.000133
PD-1	Programmed death 1	0.000469	0.001647	-0.14949	-1.10918	0.031342
PD-L1	Programmed death Ligand1	0.000413	0.001473	0.493176	1.40754	0.007822
B7-H4	coinhibitory factor for T cells	0.03157	0.047119	-0.24178	-1.18245	0.006947
Glutaminase		0.001587	0.003992	0.872126	1.830358	0.000185
Pdcd4	programmed cell death 4	9.87E-06	9.40E-05	0.554596	1.468757	0.000565
Glutamate-D1-2		2.09E-05	0.000152	0.315952	1.244833	0.00286
PDHA1	Pyruvate dehydrogenease alpha1	0.000159	0.000724	0.268412	1.204482	0.000217
GCLC	Glutamate-cysteine ligase catalytic subunit	2.33E-06	2.91E-05	0.155366	1.113704	0.009873
FGF-basic	Basic firbroblast growth factor	0.001087	0.003062	0.290716	1.223247	0.000843

Supplementary Fig 2. RPPA analysis on H1975 and H1975-OsiR residual tumors shows the alteration of immune response related. a) a list of immune-related proteins were changed in H1975-OsiR tumors vs H1975 tumors (top). Table shows the statistical significance of those changes. **b**) Bar graph showed alteration of immune related proteins in H1975 residual tumors after prolonged osimertinib treatment (top). Table shows the statistical significance of those changes.

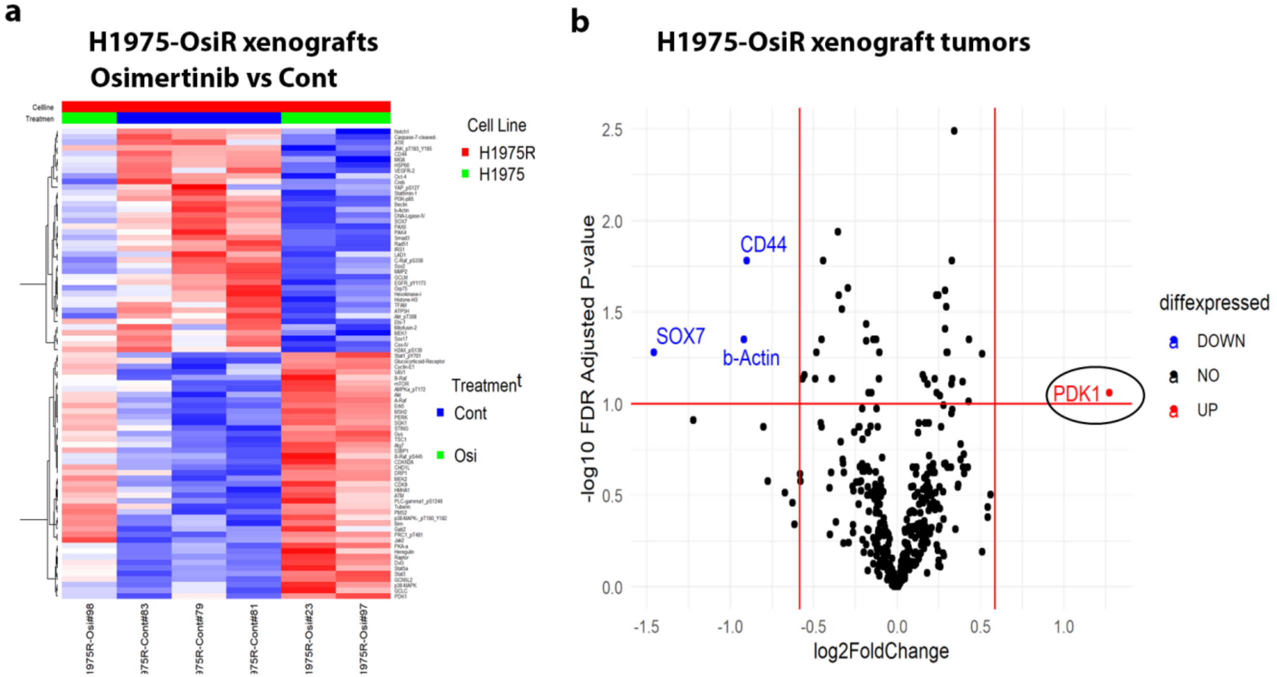

c

	ANOVA		Pairwise		
**Protein**	**p-Value**	**FDR**	**Fold Change**	**p-Value**	**FDR**
**PDK1**	**0.000**	**0.000**	**2.415**	**0.009**	**0.087**
**STING**	**0.001**	**0.003**	**1.348**	**0.002**	**0.045**
**Erk5**	**0.000**	**0.000**	**1.268**	**0.000**	**0.003**
**A-Raf**	**0.000**	**0.000**	**1.256**	**0.000**	**0.017**
**p38-MAPK-_pT180_Y182**	**0.004**	**0.009**	**1.113**	**0.005**	**0.070**
**EGFR_pY1173**	**0.002**	**0.004**	**-1.077**	**0.003**	**0.053**

Supplementary Fig 3. RPPA Gene expression profile analysis in osimertinib treated residual xenograft tumors. H1975-OsiR xenograft tumors were developed in NSG mice under continuous osimertinib treatment. RPPA data derived from the osimertinib treated residual tumors were compared with that of untreated H1975-OsiR tumors in pairwise comparison. Heatmap, Volcano plot and the list of upregulated proteins are shown in **a**, **b** and **c** respectively. The criteria of protein selection for significantly up- or down-regulation were: 1. Significant in overall F-test (FDR-adjusted p-value<0.1); 2. Significant in pairwise comparison. (FDR-adjusted p-value<0.1); 3. The Fold change of >1.5 or <-1.5 indicates whether a gene is up-regulated or down-regulated.

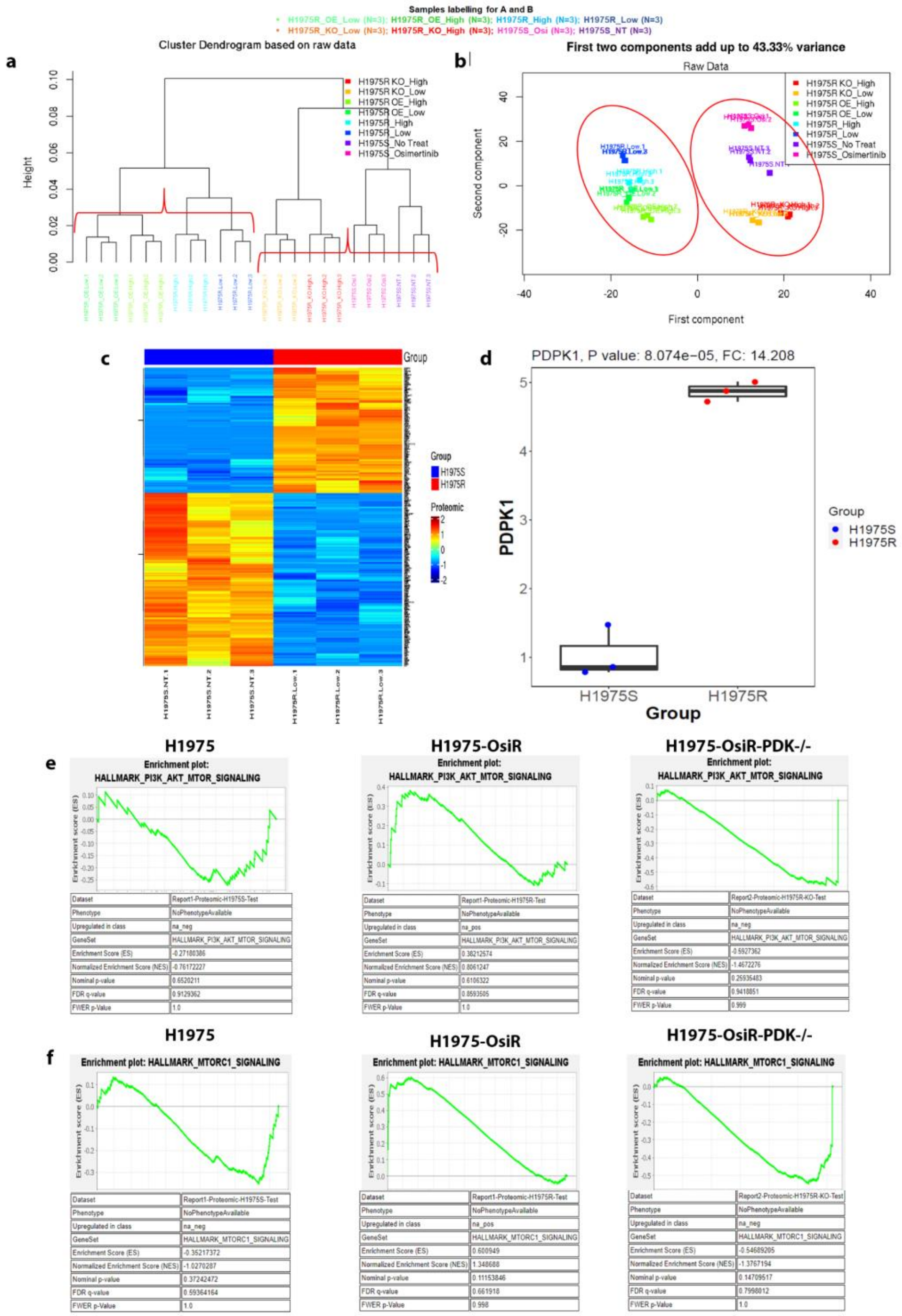

Supplementary Fig 4. Mass spectrometry-based proteomic analysis confirms upregulation of PDK1 and its downstream signaling in osimertinib resistant NCI-H1975-OsiR clones. Four isogeneic cell lines (H1975, H1975-OsiR, H1975-OsiR-PDK$^{-/-}$ & H1975-OsiR-PDK$^{++/++}$) with three biological replicates underwent Mass Spec analysis for global proteome and phospho-proteome. **a**) cluster dendrogram shows the proximity of H1975-parental and PDK knockout cells. **b**) Two component curves divide the samples into two groups which separate H1975 and PDK KO and the remaining two cell lines; Treatment “Low” is 1uM and “High” is 2.5uM osimertinib treatment **c**) Heatmap of phospho-proteins comparing H1975 and H1975-OsiR cell lines; **d**) significant upregulation of pPDK1 in H1975-OsiR cells vs H1975 cells; **e**) Protein enrichment analysis shows the difference in enrichment of PI3K/AKT/mTOR signaling among H1975, H1975-OsiR and H1975-OsiR-PDK$^{-/-}$ cells; **f**) the differences in protein enrichment of MTORC1 signaling in H1975, H1975-OsiR and H1975-OsiR-PDK$^{-/-}$ cells.

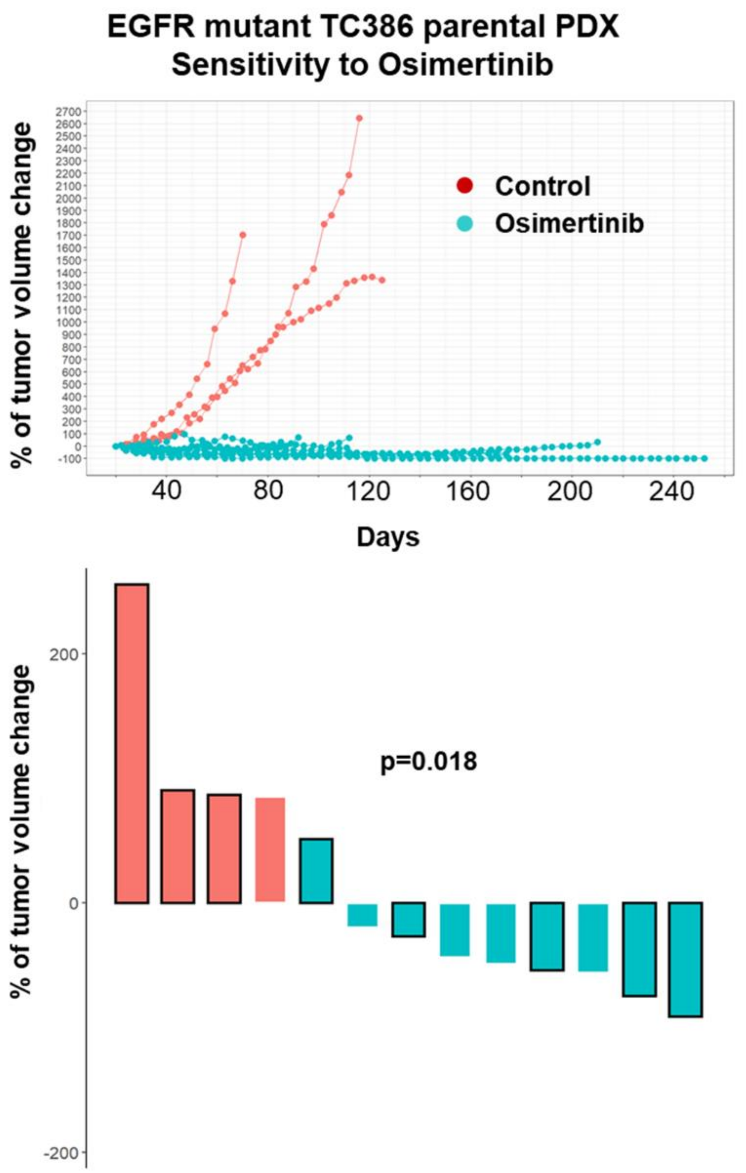

Supplementary Fig 5. Osimertinib treatment responses on EGFR mutant NSCLC TC386 PDX. Top panel: percentage of tumor volume change on individual mice with starting volume at 200 mm^3 and treatment starting at Day 0. Bottom panel: Tumor volume changes for each individual mouse at Day 21 of treatment.

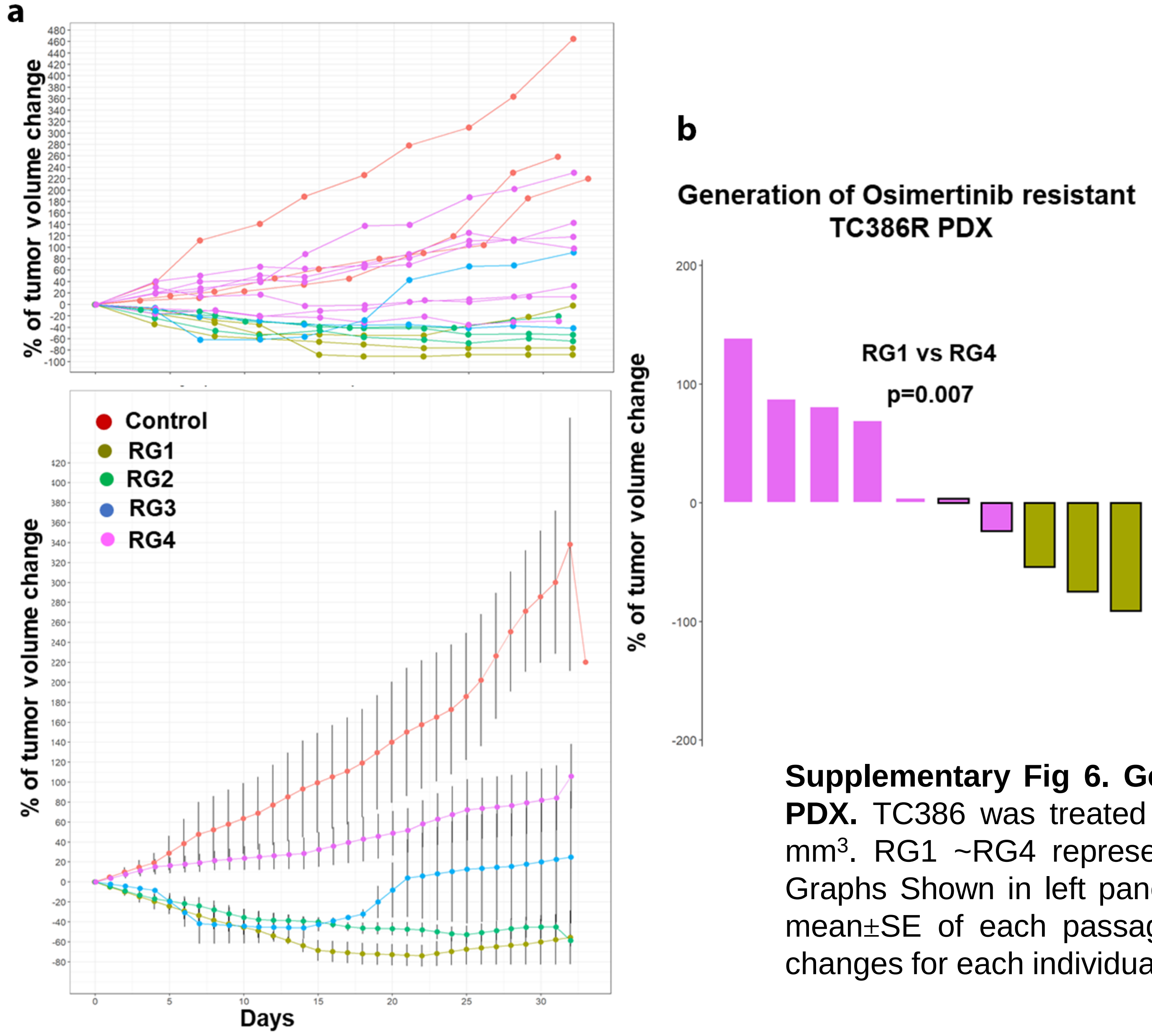

Supplementary Fig 6. Generation of osimertinib acquired resistance in TC386 PDX. TC386 was treated with osimertinib after regressed tumor grew back to 200 mm^3. RG1 ~RG4 represent 4 passages of the resistant tumors in NSG mice. **a**) Graphs Shown in left panel are tumor volume changes in the first 30 days with the mean±SE of each passage. **b**) Waterfall plot on right panel shows tumor volume changes for each individual mouse at Day 21 of treatment.

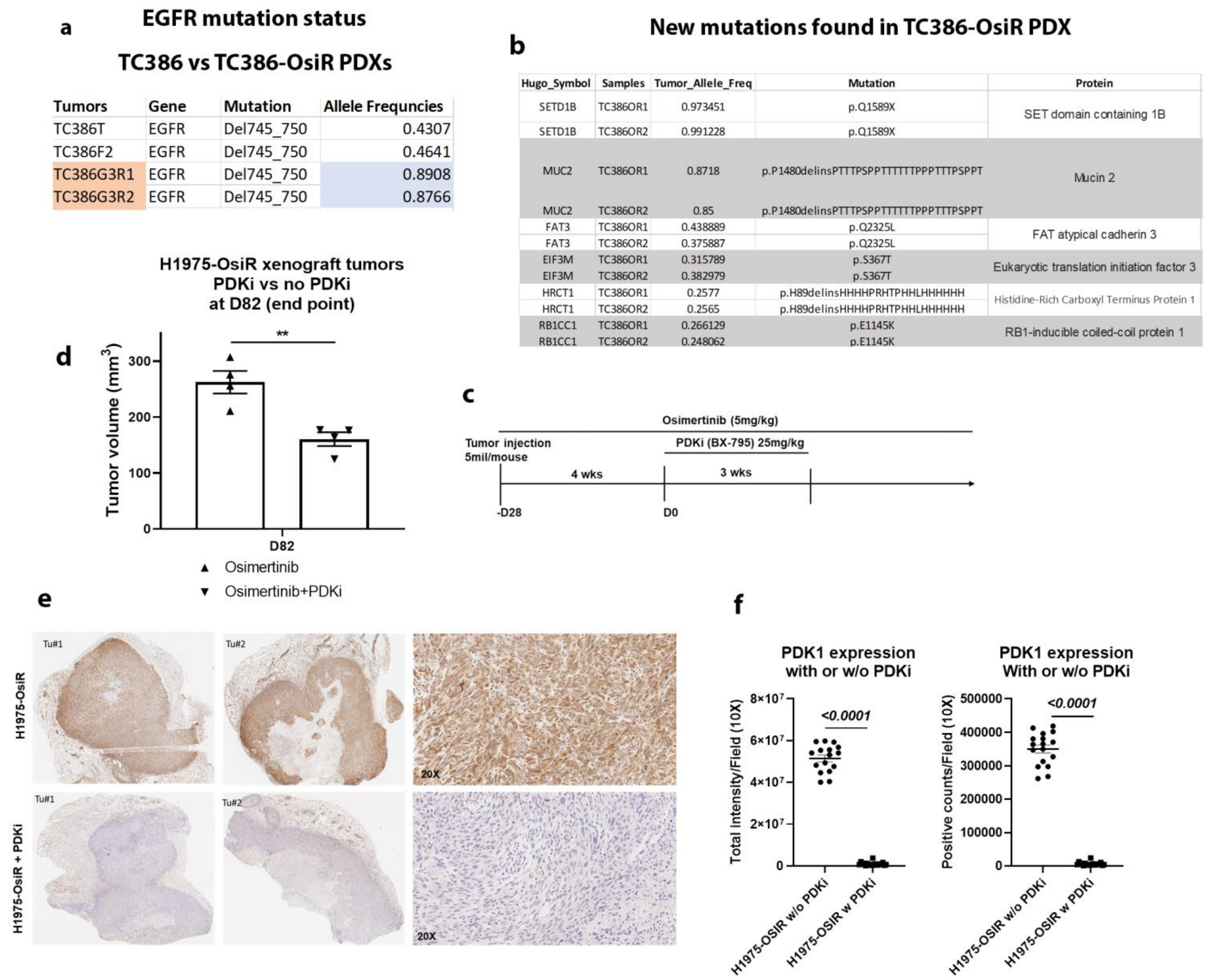

Supplementary Fig 7. Whole exome sequence of TC386 sensitive and resistant PDXs, in-vivo inhibition of PDK1 by PDKi (BX 795) on osimertinib resistant H1975-OsiR xenograft tumors. Both sensitive and resistant PDXs (TC386 & TC386-OsiR PDXs) were sequenced. **a**) EGFR mutation status between TC386 vs TC386-OsiR PDXs, **b**) the list of new mutations developed in TC386-OsiR PDX. **c**) effect of PDKi on H1975-OsiR xenograft tumors. Osimertinib resistant tumors were developed in NSG mice under continuous osimertinib treatment. The BX 795 treatment timeline is shown. **d**) Antitumor effect of osimertinib +PDKi (BX 795) on H1975-OsiR xenograft tumors; N=5 mice/group; ** p-value <0.005. **e**) at the end of the experiment, tumors were harvested for PDK1 IHC. The level of expression of PDK1 was compared between BX 795 treated and non-treated tumors; **f**) the PDK signal intensity was quantitated by using ImageScope analysis software.

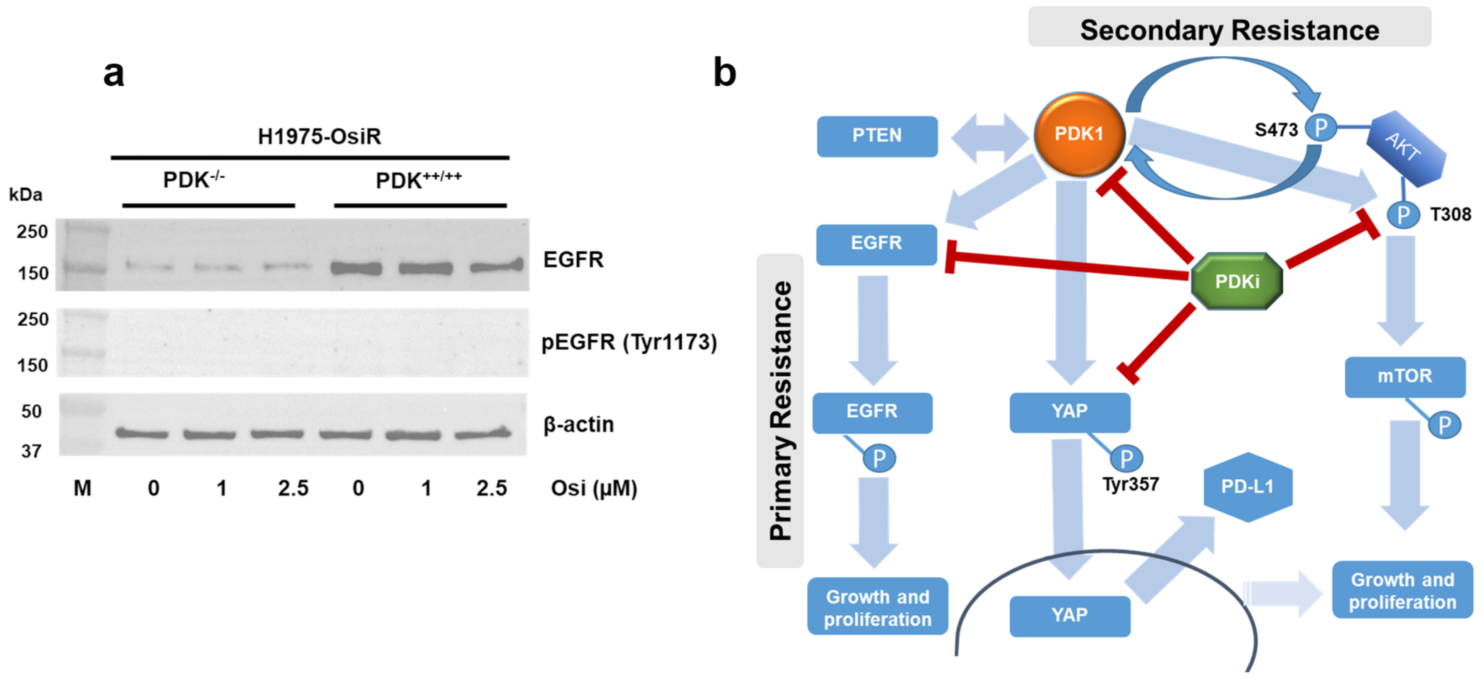

Supplementary Fig 8. a) Effect of PDK1 on EGFR and phospho-EGFR expression. H1975-OsiR-PDK$^{-/-}$ and H1975-OsiR-PDK$^{++/++}$ cells were treated with osimertinib and EGFR and pEGFR expression was analyzed by western blot. b) Graphical presentation of PDK1 role on AKT/mTOR, EGFR and YAP signaling pathways.

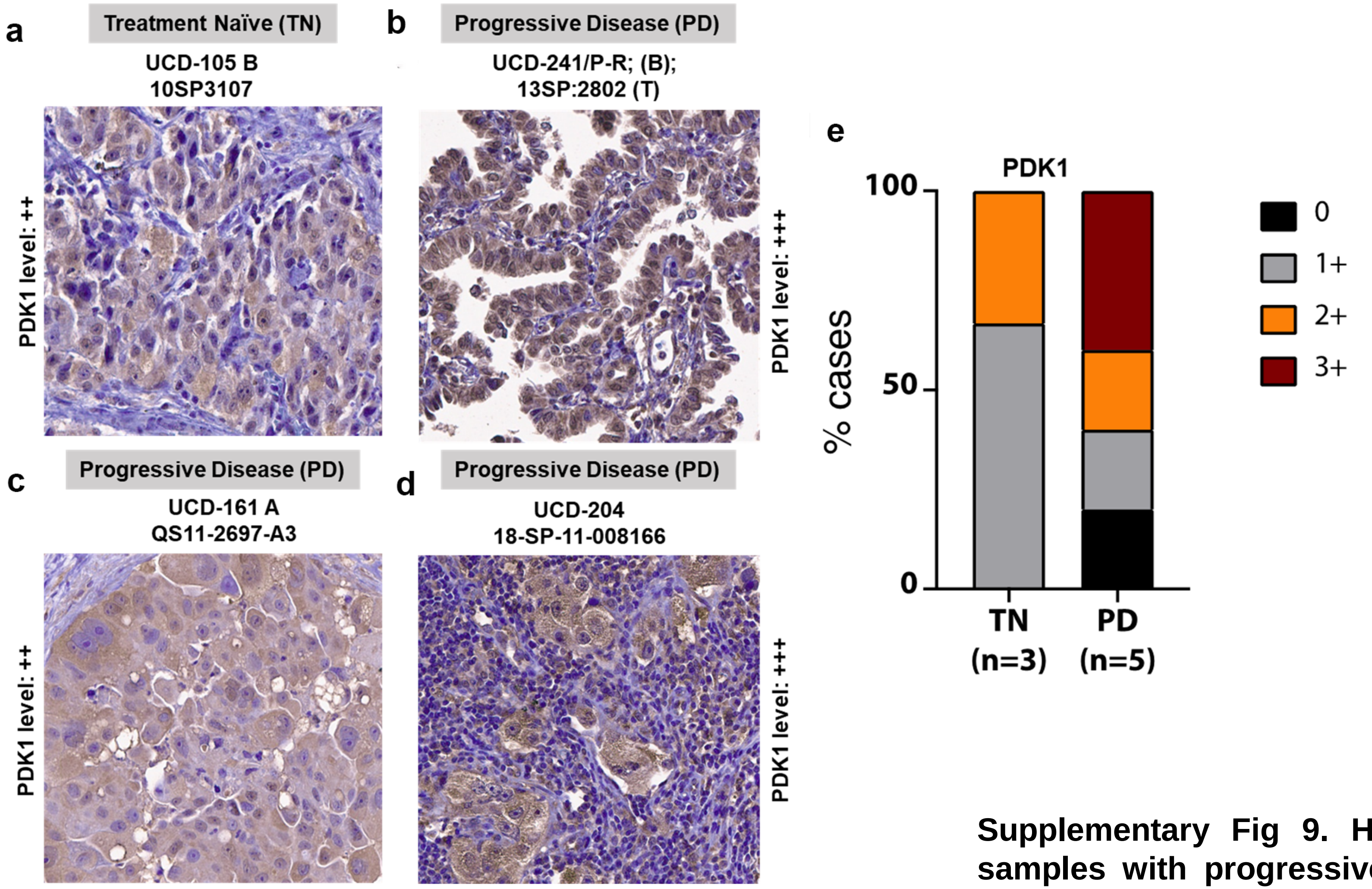

Supplementary Fig 9. Higher level of PDK1 expression in patient samples with progressive disease (PD). Immunohistochemistry (IHC) were performed in EGFR mutant treatment naïve and progressive diseases patient samples. a) Treatment Naïve (TN); EGFR E19del (E746_A750del); Stage IB NSCLC; Recurrent disease, **b)** PD; EGFR L861Q; PD on erlotinib, **c)** PD; EGFR mutant (L858R); PD on erlotinib; **d)** PD ; EGFR E19del (E746_A750del); PD on erlotinib, **e**) PDK1 IHC on TN and PD patients.

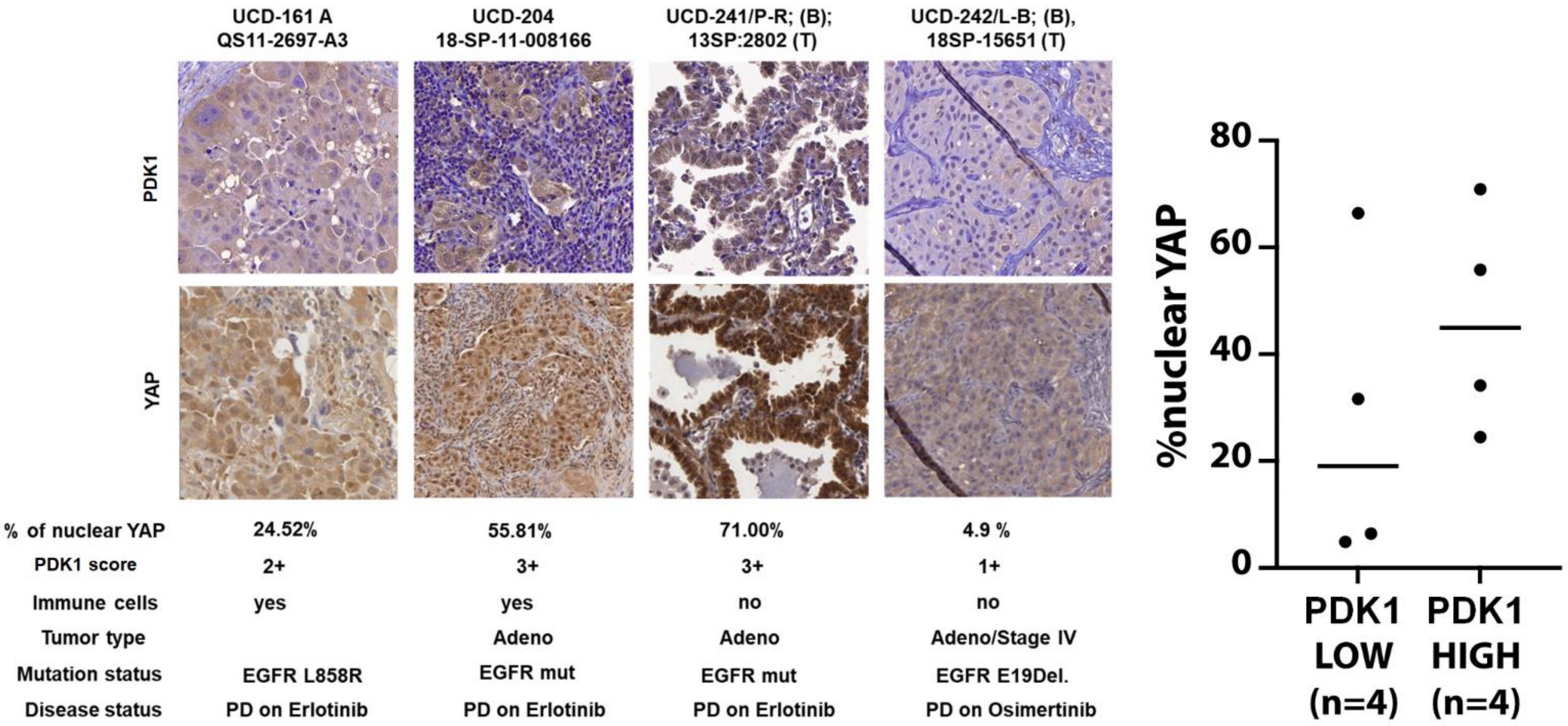

Supplementary Fig 10. Association between PDK1 expression and nuclear YAP staining in in patient samples with progressive disease (PD). Immunohistochemistry (IHC) of PDK1 and nuclear YAP were performed in EGFR mutant treatment naïve and progressive diseases patient samples. Left) Increasing percentages of nuclear YAP on high PDK1 score samples (2+, 3+ samples).Treatment Naïve (TN); EGFR E19del (E746_A750del); Stage IB NSCLC; Recurrent disease, **Right)** The level of nuclear YAP and its respective PDK1 expression.

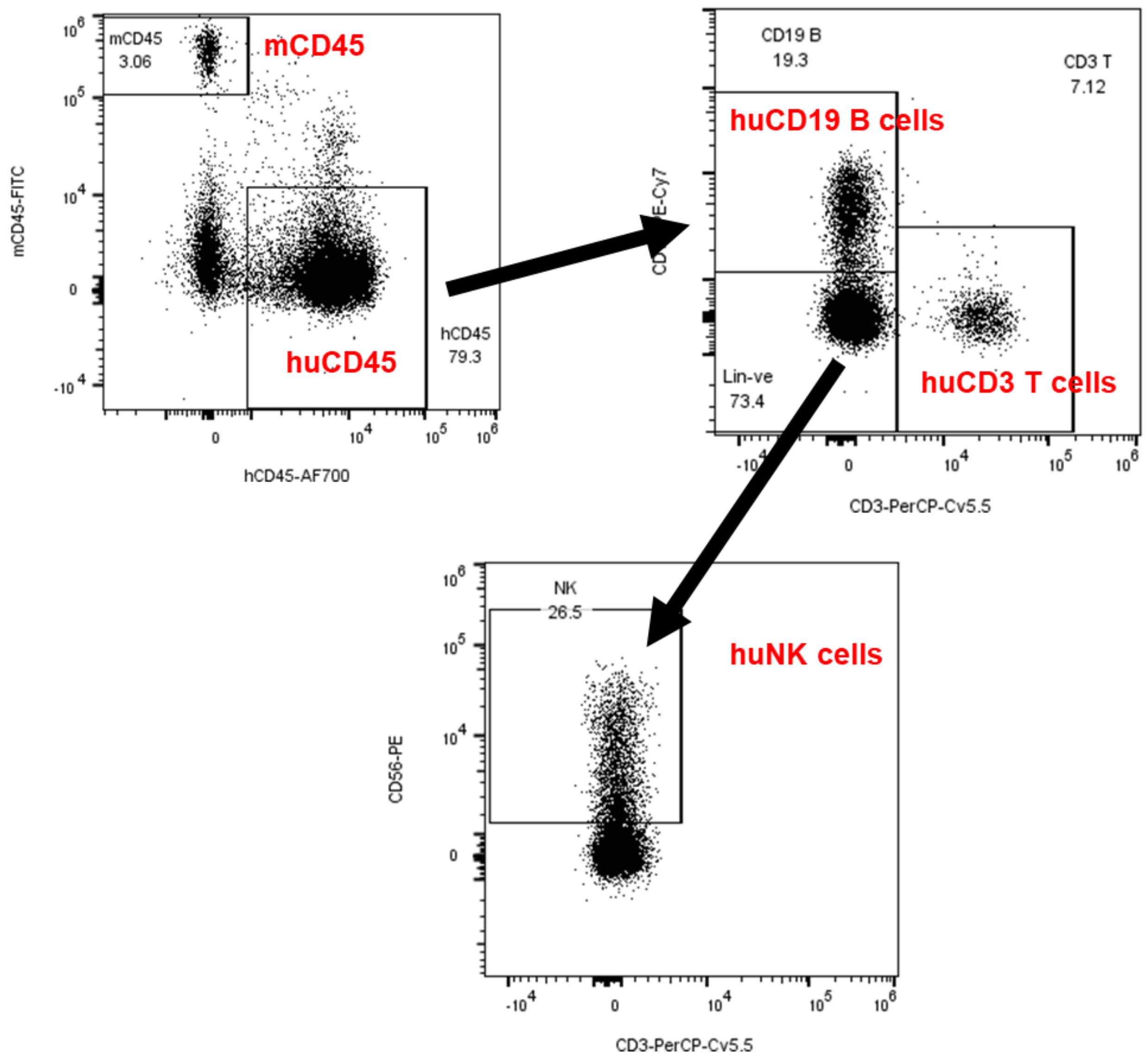

Supplementary Fig 11. Flow Cytometry gating strategy to determine the level of human immune cells in humanized mouse system

Fig 5A. Western Blot Images

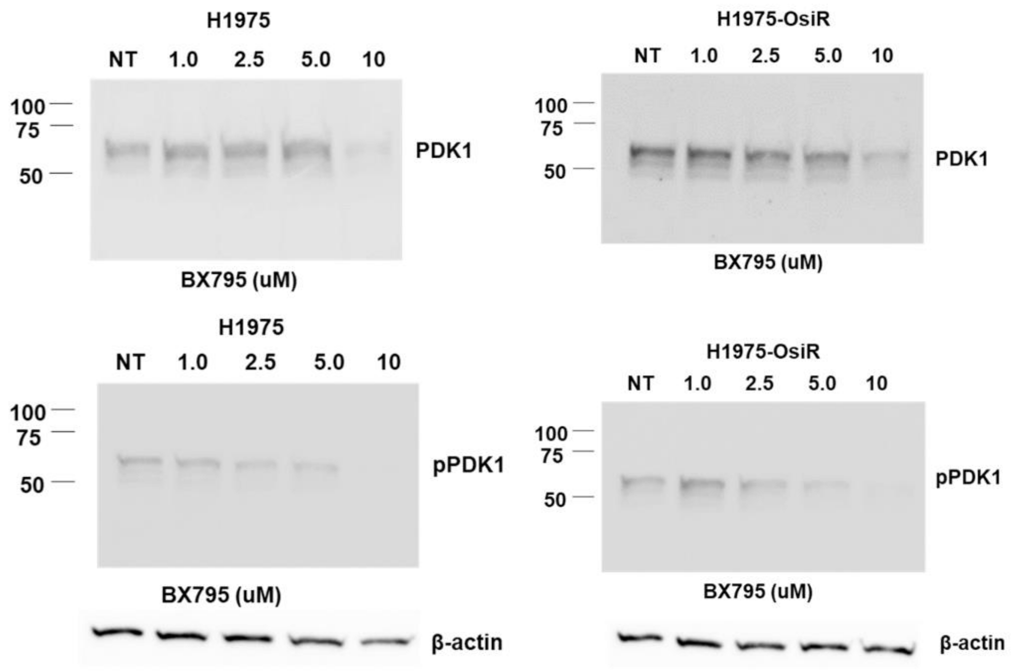

Fig 5E. Western Blot Images

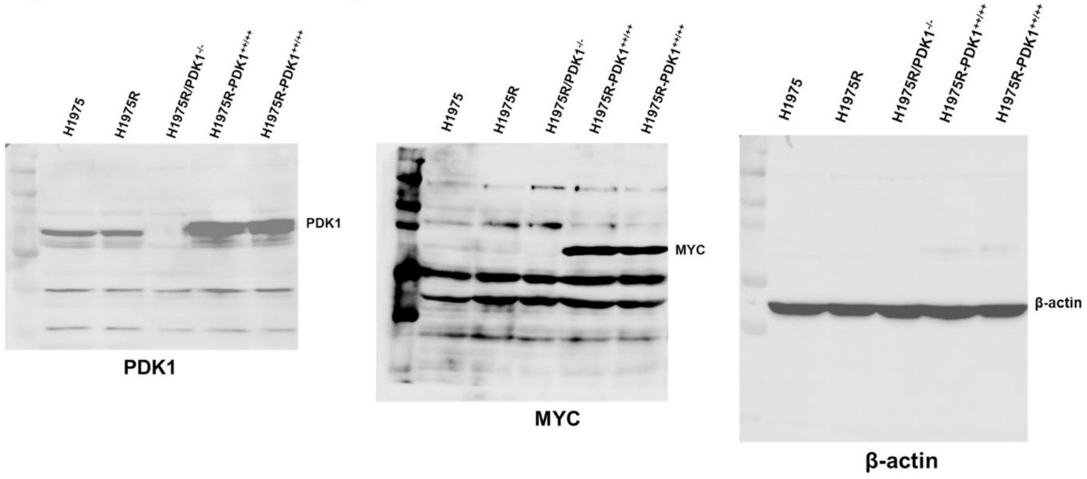

Fig 5H. Western Blot Images

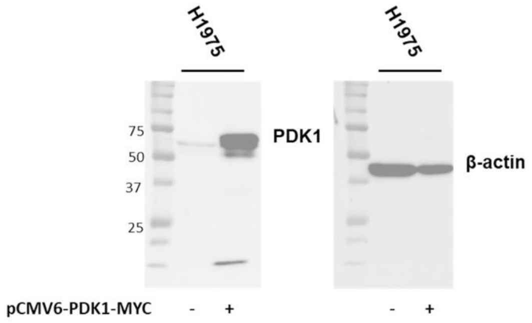

Supplementary Fig 12. Uncropped western blot images for main figure 5A, 5E and 5H

Fig 6A. Western Blot Images

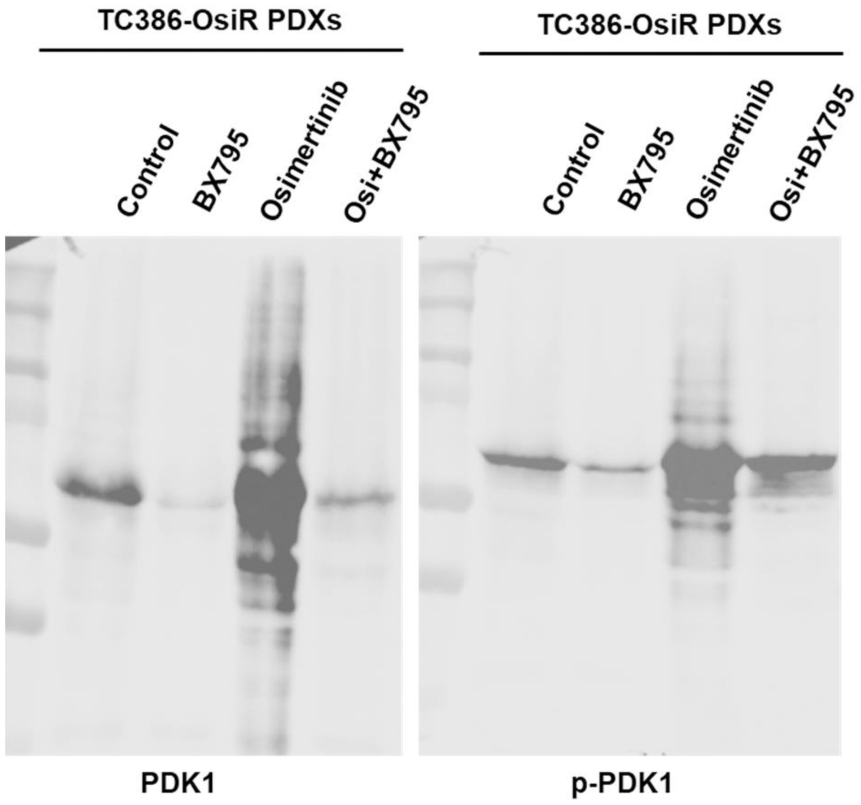

Fig 6B. Western Blot Images

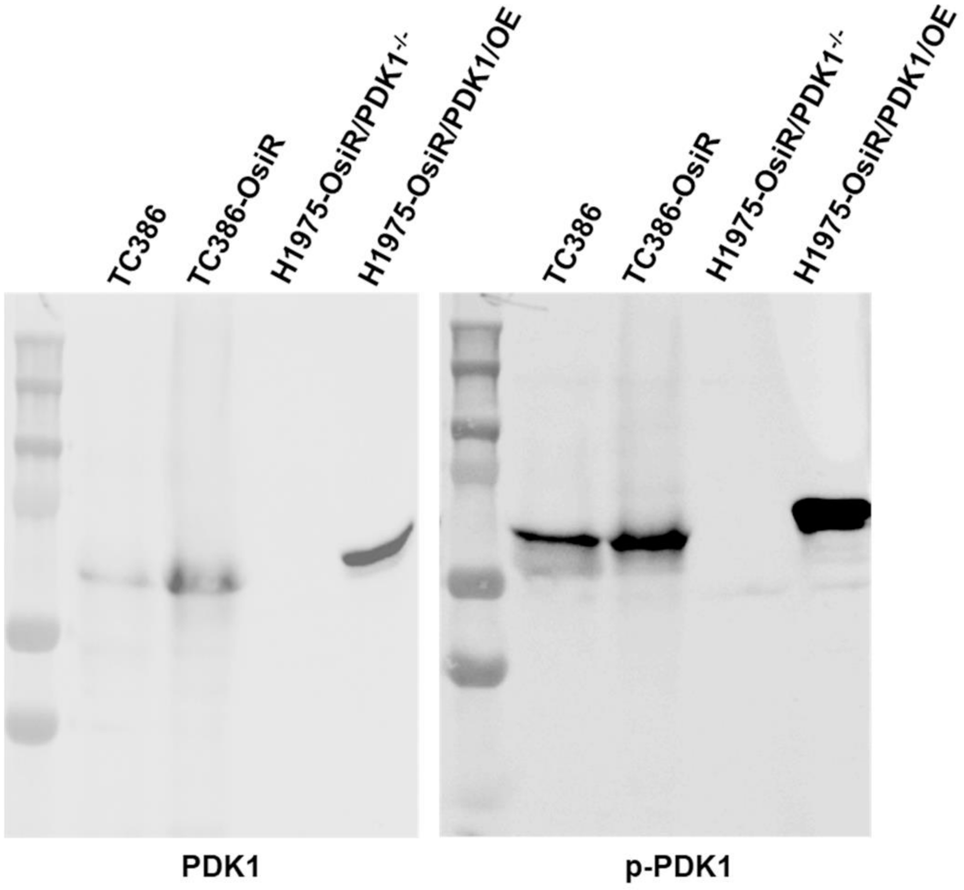

Supplementary Fig 12. Uncropped western blot images for main figure 6A-B

Fig 7A. Western Blot Images

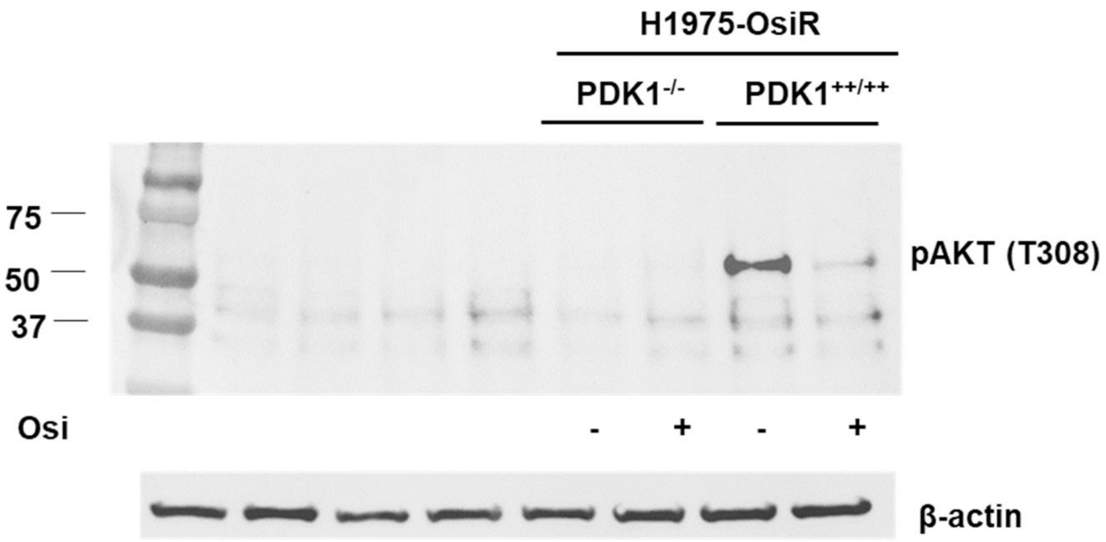

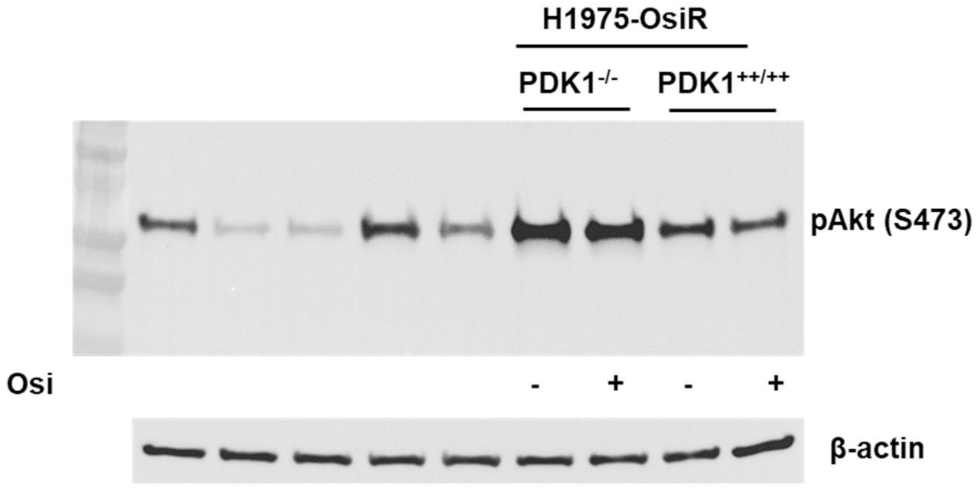

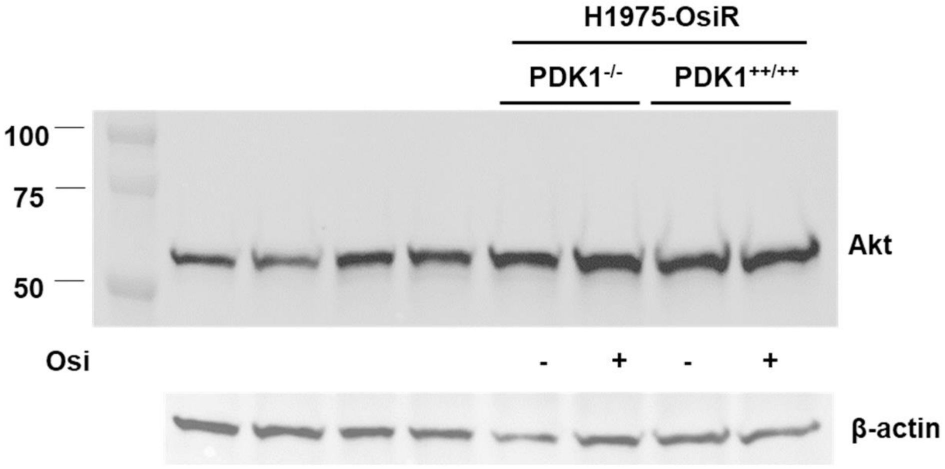

Supplementary Fig 12. Uncropped western blot images for main figure 7A

Fig 7B. Western Blot Images

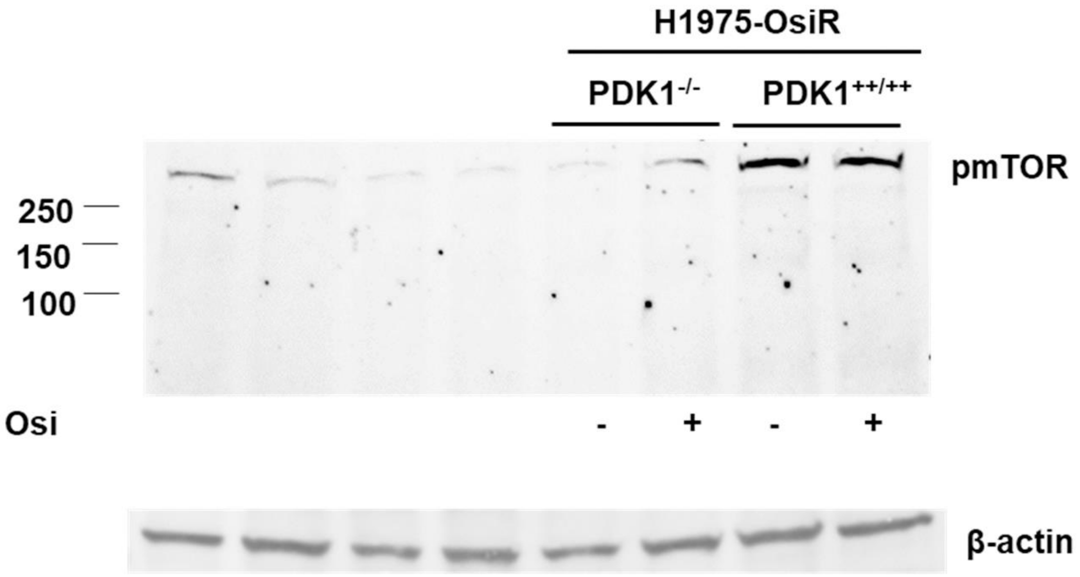

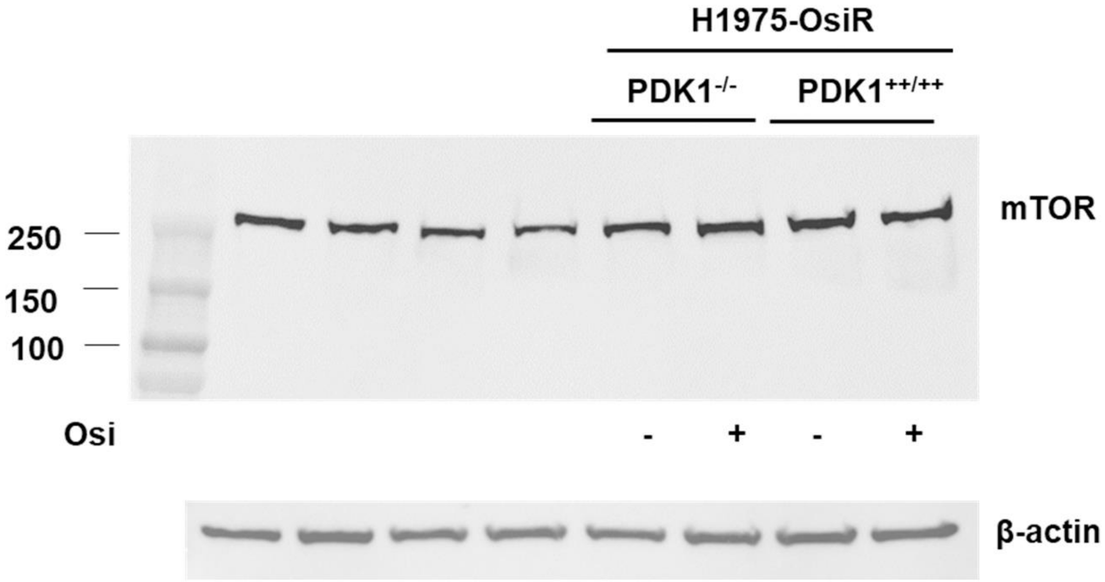

Supplementary Fig 12. Uncropped western blot images for main figure 7B

Fig 7C. Western Blot Images

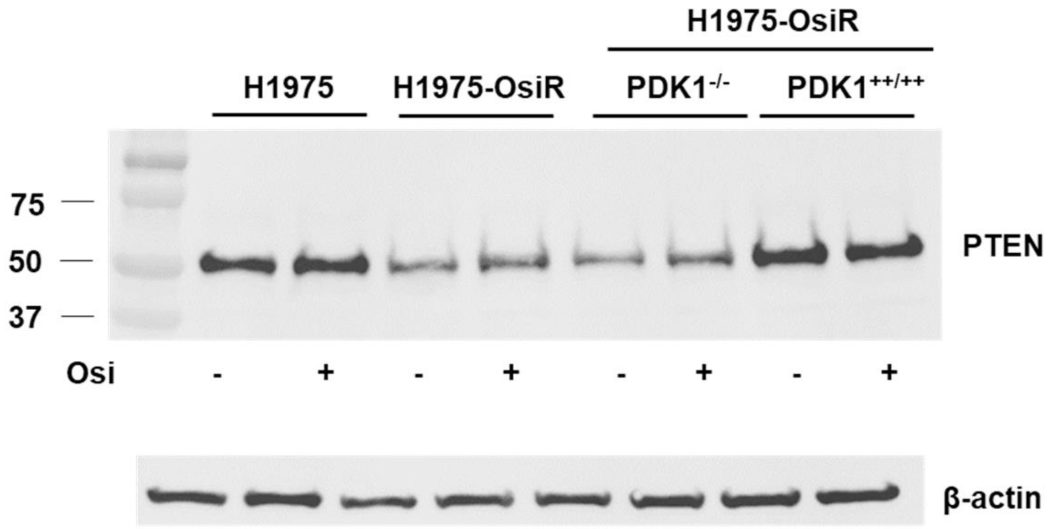

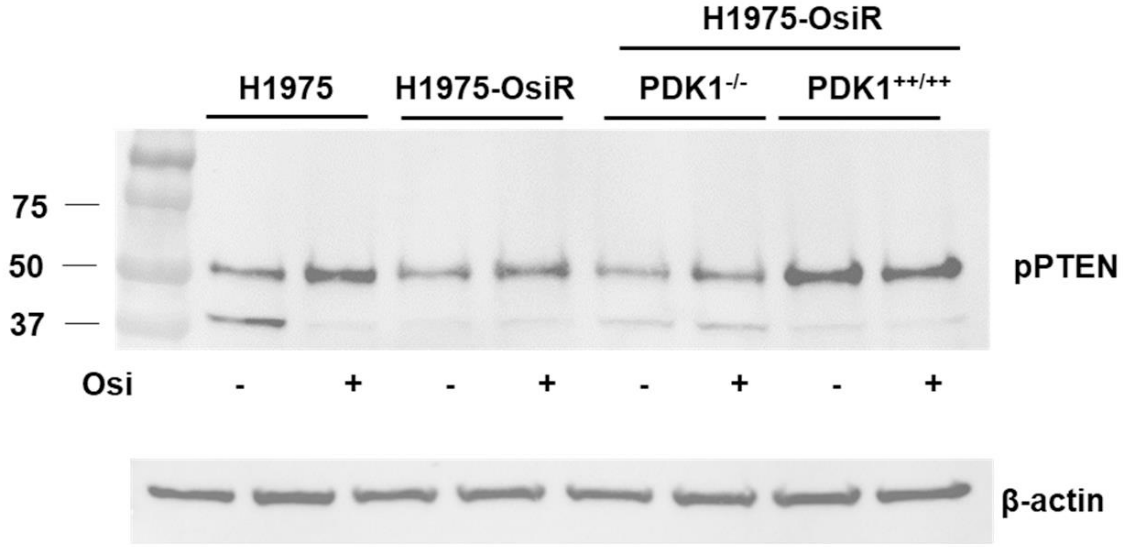

Supplementary Fig 12. Uncropped western blot images for main figure 7C

Fig 8A. Western Blot Images

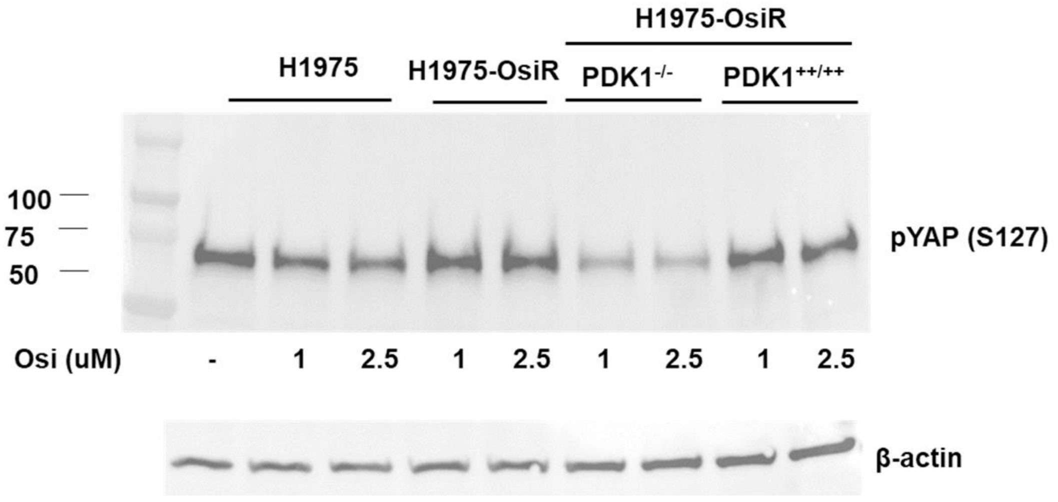

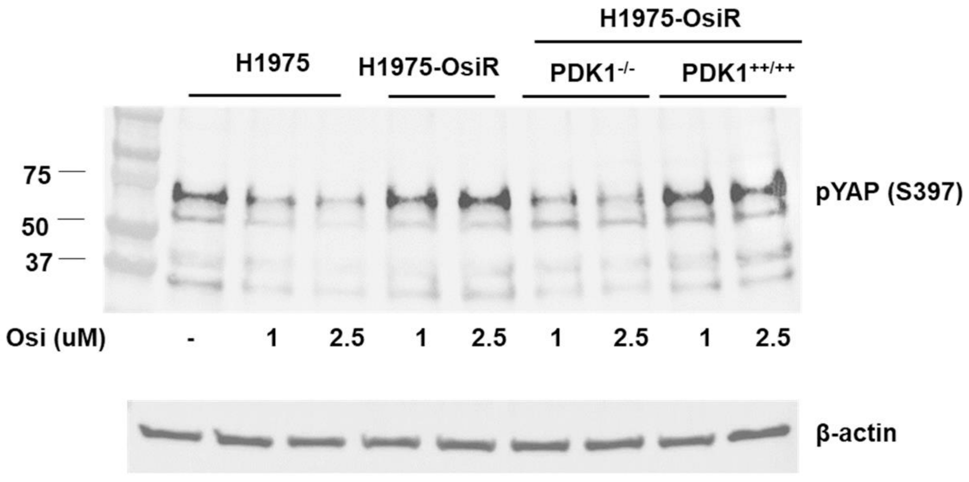

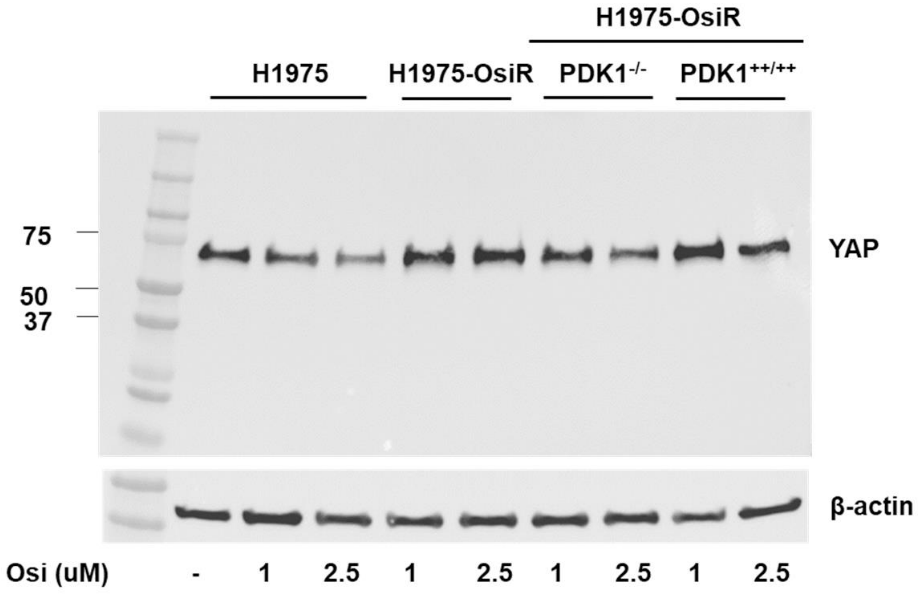

Supplementary Fig 12. Uncropped western blot images for main figure 8A

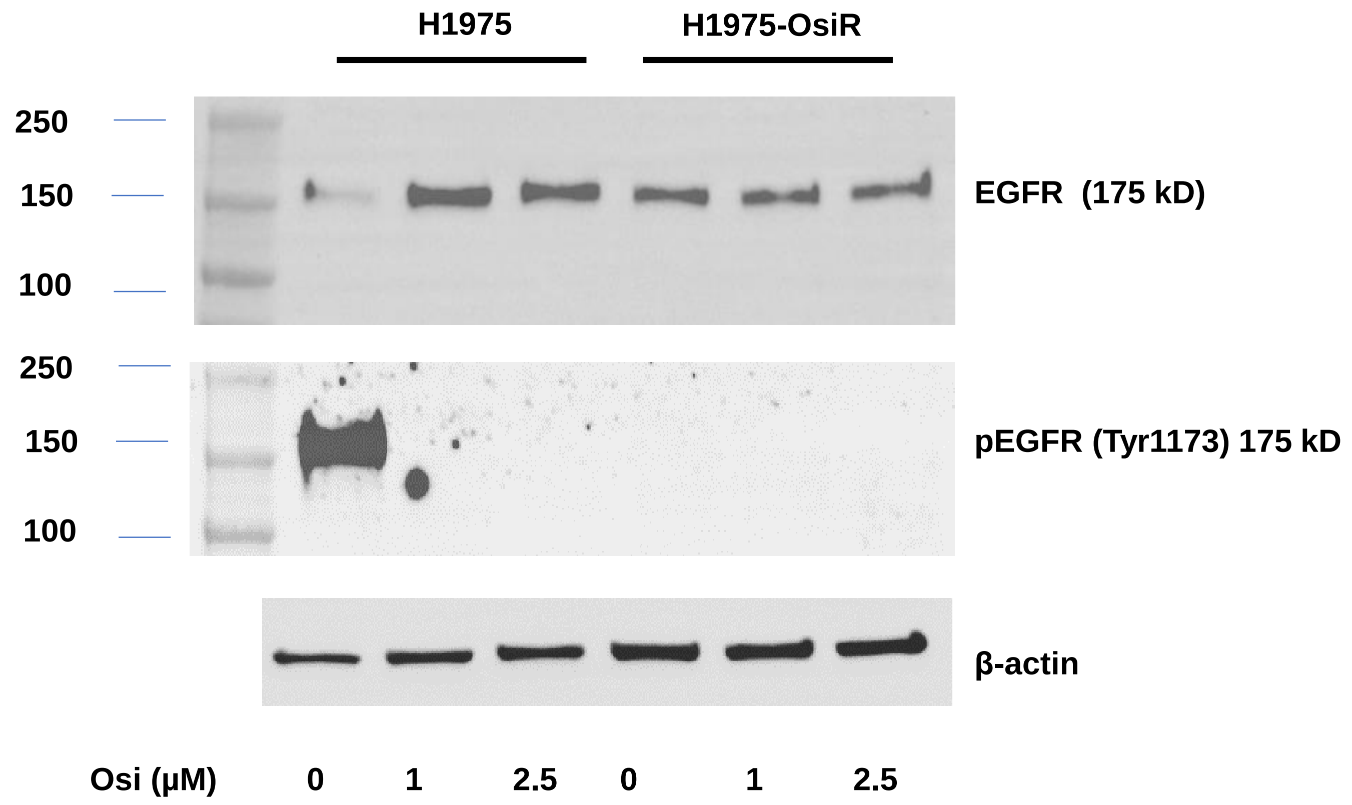

Supplementary Fig 12. Uncropped western blot images for Suppl Figure 1B

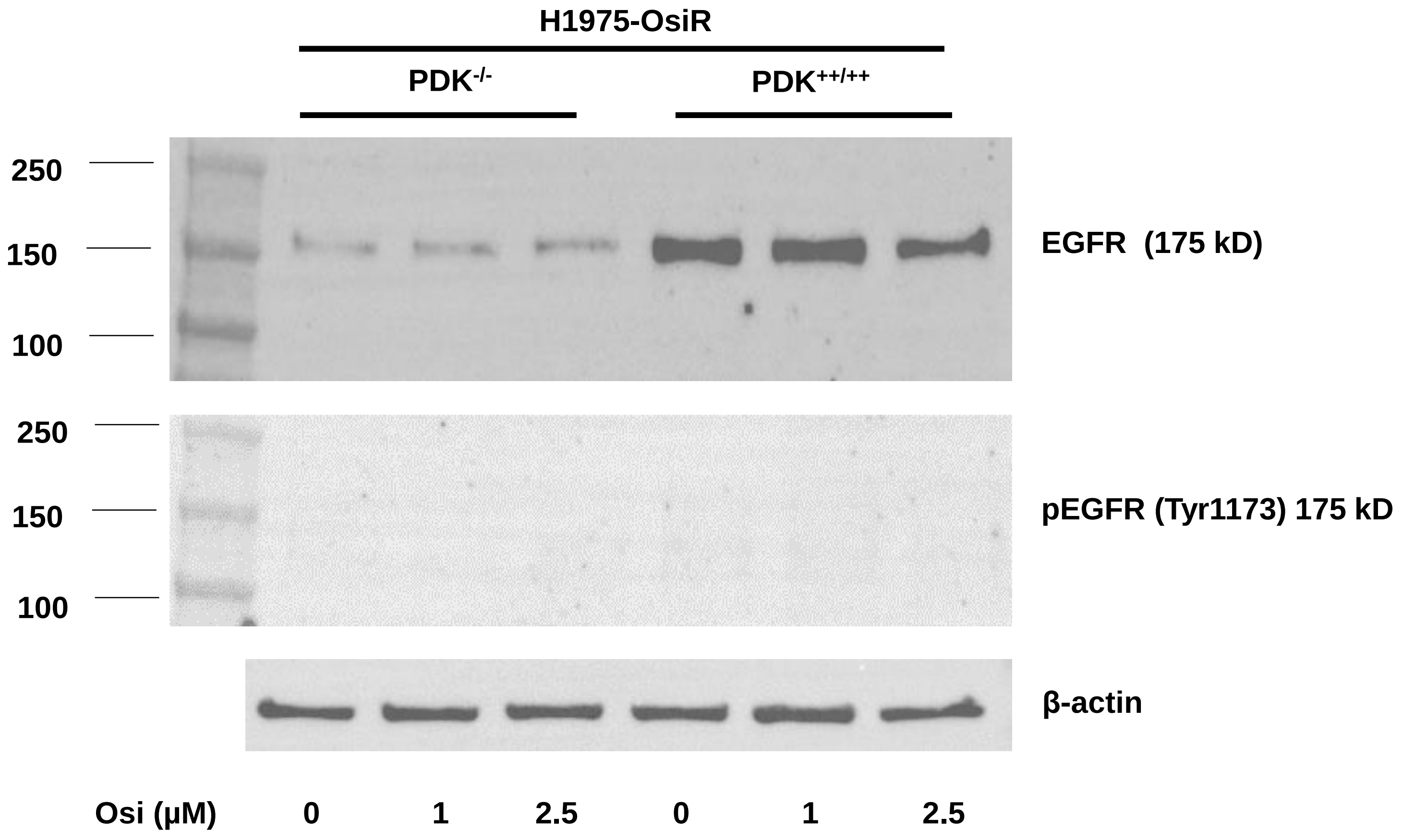

Supplementary Fig 12. Uncropped western blot images for Suppl Figure 8A
