# Supplementary material for: 3-Phosphoinositide-dependent kinase 1 drives acquired resistance to osimertinib
Source: Commun Biol. 2023 May 11;6:509. doi: 10.1038/s42003-023-04889-w (PMC10175489; doi:10.1038/s42003-023-04889-w)
Supplement: Supplementary file 2 — Description of Additional Supplementary Data [file 42003_2023_4889_MOESM2_ESM.pdf]

Description of Additional Supplementary Files

File name: Supplementary Data 1

Description: the source data behind the graphs in excel
